# Arthroscopic assisted versus open core decompression for osteonecrosis of the femoral head: A systematic review and meta-analysis

**DOI:** 10.1371/journal.pone.0313265

**Published:** 2024-11-15

**Authors:** Wensi Ouyang, Guimei Guo, Jie Xia, Changwei Zhao, Xiaoling Zhou

**Affiliations:** 1 Changchun University of Chinese Medicine, Changchun, China; 2 Hospital Affiliated to Changchun University of Traditional Chinese Medicine, Changchun, China; 3 Hunan Polytechnic of Environment and Biology, Hengyang, China; South Valley University Faculty of Medicine, EGYPT

## Abstract

**Background:**

Minimally invasive treatment options for osteonecrosis of the femoral head (ONFH) have been a prominent area of research in recent years. Arthroscopic-assisted treatments have been applied in the clinical management of ONFH; however, high-quality evidence verifying their effectiveness and safety is still lacking.

**Objective:**

To systematically assess the clinical efficacy and safety of arthroscopic-assisted core decompression (AACD) in treating ONFH.

**Methods:**

A comprehensive literature search was conducted in PubMed, Web of Science, EMBASE, Cochrane Library, Chinese National Knowledge Infrastructure, China Science and Technology Journal Database, WanFang, and the Chinese BioMedical Literature Database, from inception to June 25, 2024. We identified randomized controlled trials and non-randomized controlled studies on AACD for the treatment of ONFH based on predefined inclusion and exclusion criteria. A meta-analysis was performed using Review Manager 5.4.1 and Stata 17.0 software. The analyzed outcomes included operative time, intraoperative blood loss, length of hospital stay, postoperative femoral head collapse rate, Harris hip score, and postoperative complication rate. The Grades of Recommendations, Assessment, Development, and Evaluations (GRADE) system was used to assess the quality of evidence for the outcome indicators.

**Results:**

A total of fourteen studies were included in this meta-analysis, comprising 1,063 patients-541 in the core decompression (CD) group and 522 in the AACD group. The meta-analysis revealed no significant differences between the two groups in terms of intraoperative blood loss, length of hospital stay, 12-month postoperative Harris hip score, or overall postoperative complication rate (*P* > 0.05). However, the AACD group had a longer operative time (MD = 31.19, 95% Cl: 5.32 to 57.07, *P* = 0.02) and a lower overall postoperative femoral head collapse rate (RR = 0.49, 95% Cl: 0.27 to 0.89, *P* = 0.02) compared with the CD group. Additionally, the AACD group showed significant improvements in Harris hip scores at 3 months (MD = 6.39, 95% Cl: 5.44 to 7.33, *P* < 0.00001), 6 months (MD = 7.56, 95% Cl: 6.63 to 8.49, *P* < 0.00001), ≥ 24 months (MD = 7.00, 95% Cl: 4.80 to 9.21, *P* < 0.00001), and at the last follow-up (MD = 6.89, 95% Cl: 5.30 to 8.48, *P* < 0.00001) compared to the CD group. The GRADE evidence assessment indicated that the overall postoperative complication rate was supported by moderate-quality evidence, while the evidence for operative time, intraoperative blood loss, postoperative femoral head collapse rate, and Harris hip score was of low quality. The evidence for length of hospital stay was deemed very low quality.

**Conclusion:**

This meta-analysis suggests that AACD is an effective and safe treatment for patients with ONFH. However, due to the limited quantity and quality of the included studies, these results should be interpreted with caution. Further high-quality studies are recommended to confirm these findings.

## Trial registration

**Systematic review registration:** The study protocol has been registered in the PROSPERO database with the registration number CRD42023407838.

## Introduction

Osteonecrosis of the femoral head (ONFH) is a common orthopedic condition associated with high rates of disability and morbidity [1,2]. Its progression is relatively insidious, with intermittent hip pain and activity limitations as the primary early-stage symptoms, which significantly impact patients’ quality of life [3,4]. As the disease advances, the mechanical strength of the necrotic area weakens, leading to structural changes in the femoral head, often necessitating total hip arthroplasty [5,6]. However, total hip arthroplasty is associated with complications such as infection, prosthesis loosening, and limited longevity [7–9]. Thus, preserving the patient’s natural hip joint and delaying or even avoiding hip arthroplasty becomes particularly important. Numerous hip-preserving treatments have been documented, primarily divided into non-surgical and surgical options [10,11]. Non-surgical treatments include medications and physical therapy, which can effectively reduce pain and improve joint function but have a limited effect on halting disease progression. Studies have shown that hip-preserving surgery is often selected for ONFH patients with large necrotic lesions, involvement of the weight-bearing area, and a high risk of femoral head collapse [12,13]. Among these, core decompression (CD) is one of the most widely used clinical treatments.

The key aspect of CD is the removal of necrotic bone tissue to improve local blood circulation and promote new bone tissue formation in the necrotic region [14,15]. However, CD has its limitations, such as imprecise localization of necrotic areas and incomplete removal of necrotic lesions. With the advancement of hip arthroscopy, studies on arthroscopic-assisted core decompression (AACD) are becoming more frequent [16,17]. Through the direct visualization offered by hip arthroscopy, clinicians can more accurately assess local blood flow changes, the extent of necrosis, and the severity of lesions in the femoral head, thereby making treatment more precise and efficient [18]. Additionally, AACD offers the advantage of intraoperative diagnosis and treatment of other hip pathologies, potentially accelerating postoperative recovery [19,20]. Despite its potential, there is a lack of substantial clinical evidence supporting the efficacy of this treatment. Therefore, this study aims to systematically evaluate and analyze the clinical efficacy of AACD for the treatment of ONFH through a comprehensive systematic review and meta-analysis, providing a basis for clinical application of minimally invasive surgery in treating ONFH.

## Methods and materials

### Protocol register

This systematic review and meta-analysis adhered to the guidelines set forth in the Cochrane Handbook for Systematic Reviews and the Preferred Reporting Items for Systematic Reviews and Meta-Analyses (PRISMA) [21]. Additionally, the meta-analysis was registered in the PROSPERO database under the registration number CRD42023407838.

### Search strategy

Two reviewers (W.S.O.Y. and J.X.) conducted comprehensive searches across eight electronic databases: PubMed, Web of Science, EMBASE, Cochrane Library, Chinese National Knowledge Infrastructure, China Science and Technology Journal Database, WanFang, and Chinese BioMedical Literature Database. These searches were performed in both English and Chinese, covering the period from the databases’ inception until June 25, 2024. The search strategies incorporated keywords such as “arthroscopy”, “arthroscopic”, “surgery”, “surgical”, “operation”, “osteonecrosis of the femoral head”, “ONFH”, and “femur head necrosis”. Additionally, To ensure a thorough search, the reference lists of included articles, relevant reviews, gray literature, and key journals were also examined. Supplementary materials provide further details on the search strategy (S1 Table).

### Inclusion criteria

1. Population: The study included patients with clinically and radiologically confirmed ONFH based on diagnostic criteria such as the International Diagnostic Criteria for Osteonecrosis of the Femoral Head and the 2020 Chinese guidelines for the clinical diagnosis and treatment of osteonecrosis of the femoral head in adults [22–24]. There were no restrictions on age, gender, ethnicity, or geographical location. Staging systems utilized included the Association Research Circulation Osseous stage and the Ficat stage [25,26].
2. Interventions: Patients were treated with AACD.
3. Comparison: Patients were treated with CD.
4. Outcome measures: The outcomes assessed included operative time, intraoperative blood loss, length of hospital stay, overall postoperative femoral head collapse rate, Harris hip score, and overall postoperative complication rate.
5. Research type: Randomized controlled trials, cohort studies, and case-control studies investigating AACD as a treatment for ONFH. Only studies published in English or Chinese were included.

### Exclusion criteria

1. Studies containing overlapping data or multiple publications.
2. Reviews, conference papers, case reports, editor responses, animal experiments, basic experimental studies, technical notes, and review articles.
3. Studies with unknown or incomplete data.

### Data extraction

The literature was screened by two independent reviewers (G.M.G. and J.X.) based on the predetermined inclusion and exclusion criteria. Any disagreements were resolved through discussion, and if necessary, a third reviewer (C.W.Z.) was consulted to reach a consensus. Key study characteristics, such as first author, publication date, study design, recruited period, population details, gender ratio, mean age, number of participants and hips, stage of necrosis, mean follow-up duration, and outcome indicators, were systematically extracted by two independent reviewers (W.S.O.Y. and G.M.G.) using a structured data extraction template. Key outcomes were also extracted by two additional reviewers (C.W.Z. and X.L.Z.) for data synthesis. Any discrepancies in data extraction were addressed collaboratively through consensus among all reviewers. We have extracted all data needed for this analysis, therefore, we did not need to handle missing data in this study.

### Assessment of literature quality

Two independent reviewers (W.S.O.Y. and J.X.) assessed the methodological quality of the included studies. For randomized controlled trials, the quality was evaluated using the Cochrane Risk of Bias tool, focusing on seven key criteria: random sequence generation, allocation concealment, blinding of participants and personnel, blinding of outcome assessment, completeness of outcome data, selective reporting, and other potential biases [27]. Non-randomized controlled trials were evaluated using the Newcastle-Ottawa Scale (NOS), which assessed factors such as study population selection, group comparability, and outcome evaluation [28].

### Quality of evidence assessment

The quality of evidence for each outcome was evaluated using the Grades of Recommendations, Assessment, Development, and Evaluations (GRADE) system [29]. This system assesses the certainty of evidence across six key domains: study design limitations, risk of bias, inconsistency of results, indirectness, imprecision, and publication bias [30]. Two independent reviewers (G.M.G. and J.X.) assessed each domain for every outcome. In cases of disagreement, a third reviewer (C.W.Z.) was consulted to resolve the issue. All decisions regarding the upgrading or downgrading of the evidence’s certainty were documented to ensure transparency.

### Statistical analysis

Statistical analysis was performed using Review Manager 5.4.1 software (Cochrane Collaboration, Oxford, UK) and Stata 17.0 software (StataCorp, College Station, USA). For binary data, the risk ratio (RR) was used, while the mean difference (MD) was employed for continuous data. Both measures were reported with 95% confidence intervals (CI) to provide a range of effect sizes when comparing intervention and control groups. Heterogeneity was assessed using the chi-squared (χ^2^) test and the *I*^2^ statistic. An *I*^2^ value below 50% indicated no significant heterogeneity, justifying the use of a fixed-effects model. An *I*^2^ value above 50% suggested significant heterogeneity, necessitating the use of a random-effects model. Sensitivity analysis was performed by systematically excluding one study at a time to assess the robustness of the results. Publication bias was evaluated using a funnel plot and Egger’s test.

## Results

### Search selection

**Fig 1:**
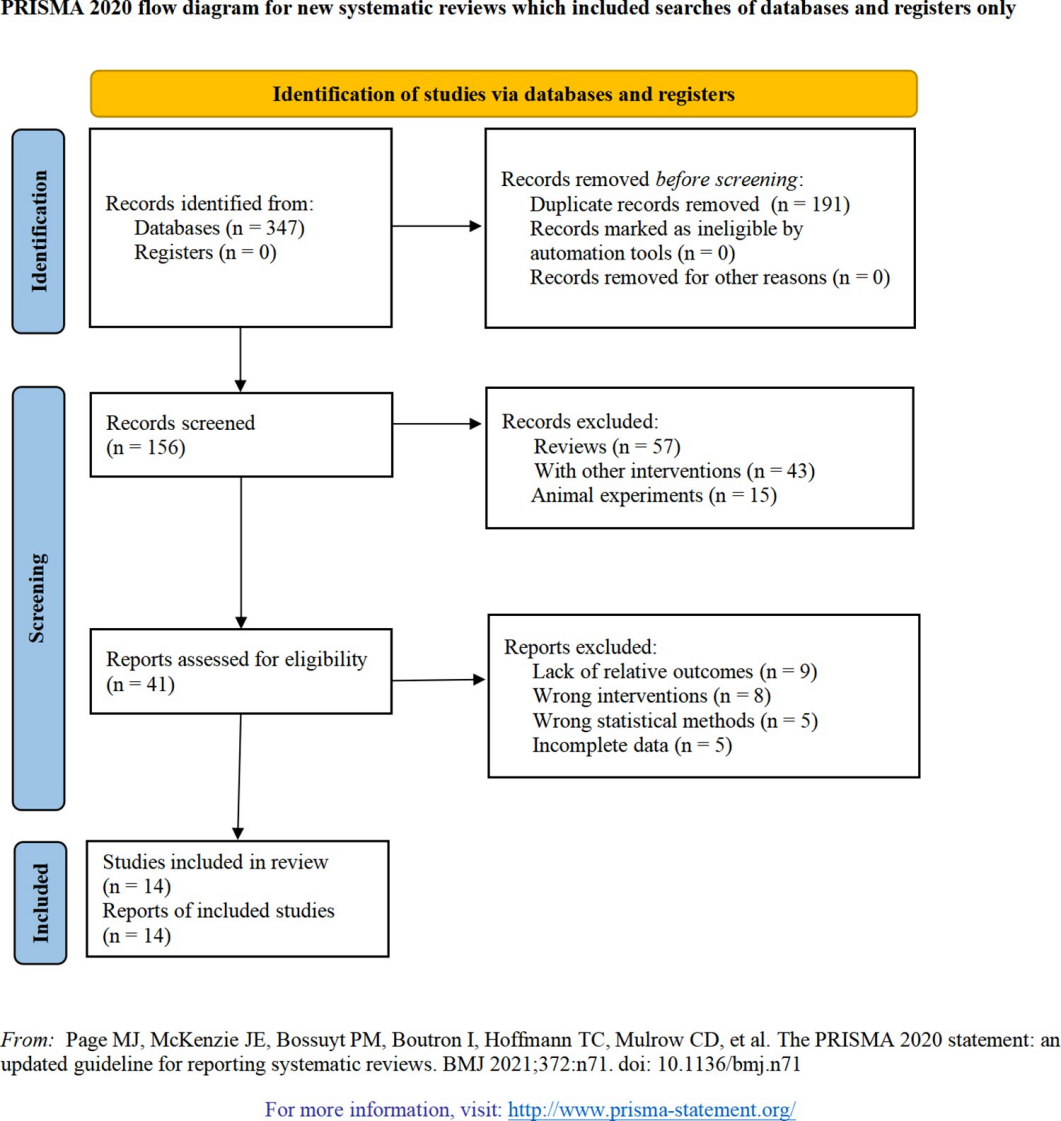
Literature screening process and results.

The initial database search yielded 347 articles on AACD treatment for ONFH. After removing 191 duplicate records, 115 articles were excluded based on their titles and abstracts. A further 27 articles were excluded after a full-text review based on the inclusion and exclusion criteria. Ultimately, 14 published articles [31–44] were included in the meta-analysis (Fig 1, S1 File). PRISMA checklist is shown in the S2 Table.

### Baseline characteristics

A total of 1,063 adult participants with ONFH were included in the 14 eligible studies [31–44]. Of these, six were randomized controlled trials [34,36,39–40,42,44], six were retrospective cohort studies [31–33,35,38,43], and two were retrospective case-control studies [37,41]. The CD group comprised 541 participants, while the AACD group included 522 participants. The follow-up period for patients ranged from 3 to 168 months. All studies included participants aged 30 years or older, with clearly defined inclusion and exclusion criteria. No significant differences in baseline characteristics were observed between the two groups. Two diagnostic standards were applied across all studies: ten studies [31–37,39,41–42] used the ARCO classification, while four studies [38,40,43–44] reported data based on the Ficat classification (Tables 1 and S3).

**Table 1:** Basic characteristics of the fourteenth studies included in the meta-analysis.

Inclusion studies	Study design	Recruited period	Sample (M/F)	Hip (M/F)	Age (years)	BMI (Kg/m^2^)	Diagnostic standard	Disease stage	Outcomes	Follow-up (months)
Yang 2024 [31]	Retrospective cohort study	2013.3- 2018.12	A: 18 (16/2) C: 21 (18/3)	A: NA C: NA	A: 39.7 ± 8.5 C: 40.8 ± 10.2	A: 22.8 ± 2.9 C: 21.6 ± 2.9	ARCO	Ⅱ/Ⅲ	④⑤	A: 38.3 ± 18.9 C: 34.6 ± 8.2
Zhao 2024 [32]	Retrospective cohort study	1998.2- 2012.12	A: 92 (54/38) C: 68 (39/39)	A: NA C: NA	A: 38.21 ± 8.73 C: 41.31 ± 10.24	A: 25.19 ± 3.38 C: 24.98 ± 4.13	ARCO	Ⅱ/Ⅲ	④⑤	168
Zhao 2023 [33]	Retrospective cohort study	2015.1- 2019.12	A: 41 (25/16) C: 60 (34/26)	A: 59 (37/22) C: 80 (48/32)	A: 35.5 ± 9.8 C: 37.7 ± 10.5	A: 23.5 ± 2.3 C: 22.9 ± 12.6	ARCO	Ⅰ/Ⅱ	①②③④⑤	12
Lian 2021 [34]	Randomized controlled trial	2018.1- 2020.6	A: 48 (22/26) C: 48 (23/25)	NA	A: 40.25 ± 5.11 C: 40.98 ± 5.83	NA	ARCO	Ⅱ/Ⅲ	①②③⑤⑥	6
Dou 2020 [35]	Retrospective cohort study	2016.1- 2018.12	A: 23 (13/10) C: 29 (16/13)	A: 31 (NA) C: 36 (NA)	A: 38.52 ± 7.38 C: 36.58 ± 6.91	A: 22.51 ± 4.86 C: 23.41 ± 6.93	ARCO	Ⅰ/Ⅱ	①③④⑤⑥	14.95 ± 1.57
Zhang 2020 [36]	Randomized controlled trial	2018.1- 2019.2	A: 42 (27/15) C: 42 (29/13)	A: NA C: NA	A: 41.65 ± 8.42 C: 41.28 ± 8.74	NA	ARCO	Ⅱ/Ⅲ	①②③⑤	3
Li 2017 [37]	Retrospective case control study	2006.4- 2010.11	A: 26 (15/11) C: 34 (18/16)	A: 43 (NA) C: 55 (NA)	A: 37.4 ± 10.3 C: 35.2 ± 11.8	NA	ARCO	Ⅰ/Ⅱ	④⑤	A: 61.4 ± 5.7 C: 53.9 ± 4.1
Li 2017 [38]	Retrospective cohort study	2010.3- 2013.12	A: 39 (26/13) C: 52 (32/20)	A: 53 (36/17) C: 74 (46/28)	A: 32.7 C: 31.3	NA	Ficat	Ⅰ/Ⅱ	①④⑤⑥	A: 39.3 C: 34.6
Zhuang 2017 [39]	Randomized controlled trial	2012.4- 2013.4	A: 57 (NA) C: 65 (NA)	A: 57 (NA) C: 65 (NA)	NA	NA	ARCO	Ⅱ/Ⅲ	①②⑤⑥	3
Liu 2015 [40]	Randomized controlled trial	2013.1- 2014.6	A: 39 (28/11) C: 32 (17/15)	A: 44 (32/12) C: 37 (19/18)	A: 41.36 ± 11.74 C: 40.21 ± 9.25	NA	Ficat	Ⅰ/Ⅱ/Ⅲ	⑤	6
Wu 2015 [41]	Retrospective case control study	2011.2- 2013.2	A: 39 (20/19) C: 40 (22/18)	NA	A: 38.35 ± 10.15 C: 37.01 ± 11.25	NA	ARCO	Ⅱ	⑤	24
Liu 2013 [42]	Randomized controlled trial	2008.3- 2009.4	A: 17 (NA) C: 15 (NA)	A: 20 (NA) C: 20 (NA)	A: 36.5 C: 36.5	NA	ARCO	Ⅰ/Ⅱ	⑤	48.7
Zhuo 2012 [43]	Retrospective cohort study	2007.1- 2010.3	A: 18 (11/7) C: 10 (5/5)	A: 21 (12/9) C: 12 (6/6)	A: 32.5 C: 30.8	NA	Ficat	Ⅰ/Ⅱ	④⑤	30
Han 2008 [44]	Randomized controlled trial	2005.3- 2008.4	A: 23 (NA) C: 25 (NA)	A: 30 (NA) C: 30 (NA)	A: 33.6 C: 33.6	NA	Ficat	0/Ⅰ/Ⅱ	⑤	32.3

**Note:** A, Arthroscopic-assisted core decompression group; ARCO, Association Research Circulation Osseous; BMI, Body mass index; C, Core decompression group; NA, not available. Outcome Indicator: ① operative time, ② intraoperative blood loss, ③ length of hospital stay, ④ overall postoperative femoral head collapse rate, ⑤ Harris hip score, ⑥ overall postoperative complication rate.

### Risk of bias assessment

**Fig 2:**
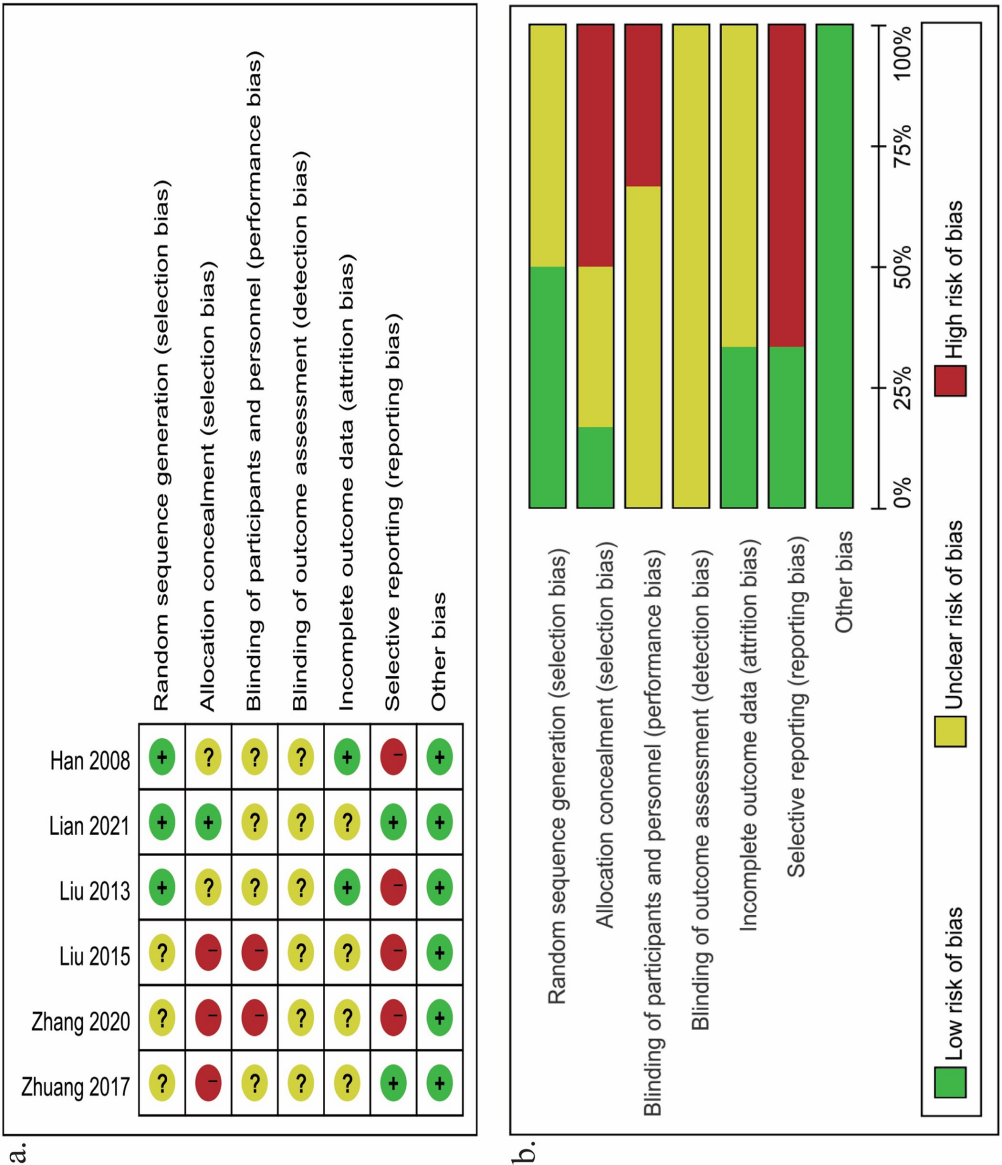
Risk of bias graph in the included studies: A Risk of bias summary b Risk of bias graph.

The risk of bias for each included randomized controlled trial is presented in Fig 2. The six retrospective cohort studies [31–33,35,38,43], and two retrospective case-control studies [37,41] were evaluated using the NOS, with all studies rated as high quality, achieving scores ranging from 7 to 8, as detailed in S4–S6 Tables.

### Meta-analysis results

#### Operative time

**Fig 3:**
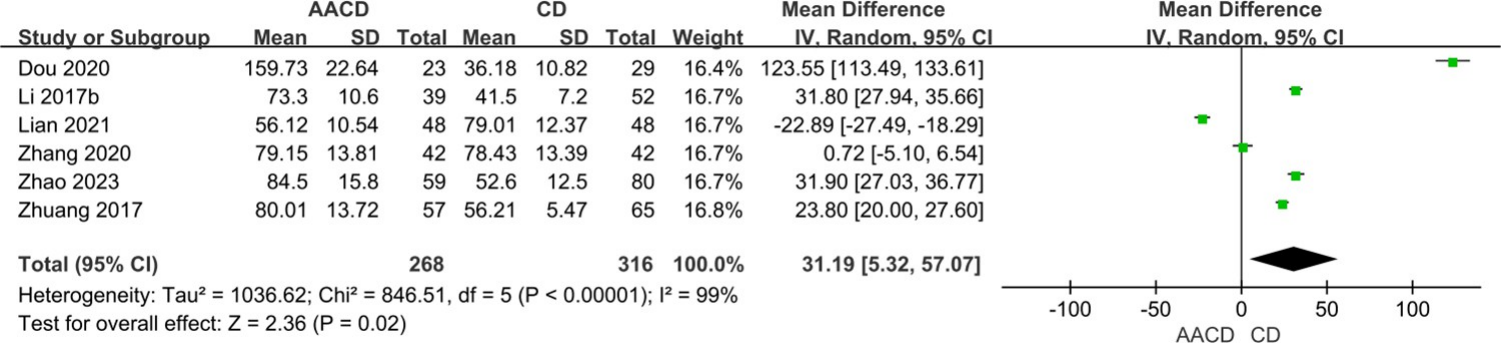
Forest plot of the meta-analysis comparing the operative time.

Six studies [33–36,38,39] with a total of 584 participants reported on operative time. Heterogeneity was observed among the studies (*P* < 0.00001, *I*^2^ = 99%), leading to the selection of a random-effects model. The results demonstrated a significant difference in operative time between the two groups (MD = 31.19, 95% Cl: 5.32 to 57.07, *P* = 0.02) (Fig 3, Table 3).

#### Intraoperative blood loss

**Fig 4:**
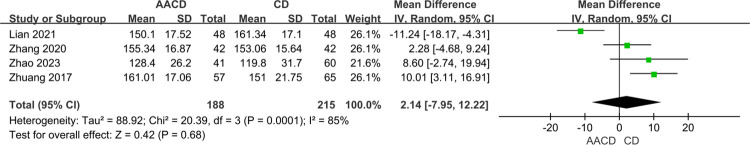
Forest plot of the meta-analysis comparing the intraoperative blood loss.

Four studies [33–34,36,39] involving 403 participants reported on intraoperative blood loss. Heterogeneity was present (*P* = 0.0001, *I*^*2*^ = 85%), so a random-effects model was used. The analysis showed no significant difference in intraoperative blood loss between the two groups (MD = 2.14, 95% Cl: -7.95 to 12.22, *P* = 0.68) (Fig 4, Table 3).

### Length of hospital stay

**Fig 5:**
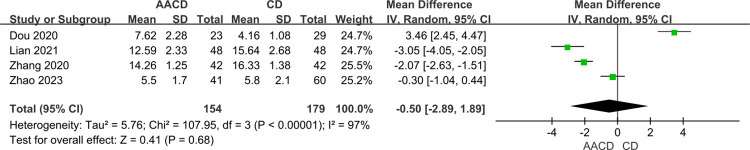
Forest plot of the meta-analysis comparing the length of hospital stay.

Four studies [33–36] with 333 participants reported on the length of hospital stay. Significant heterogeneity was noted (*P* < 0.00001, *I*^*2*^ = 97%), prompting the use of a random-effects model. The results indicated no significant difference in the length of hospital stay between the two groups (MD = -0.50, 95% Cl: -2.89 to 1.89, *P* = 0.68) (Fig 5, Table 3).

#### Overall postoperative femoral head collapse rate

**Fig 6:**
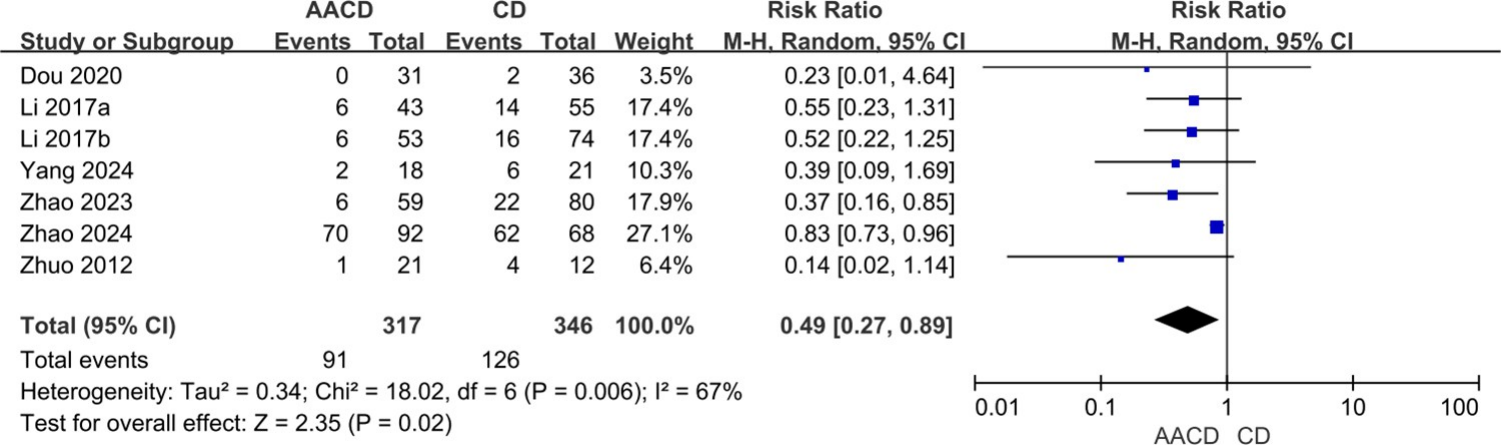
Forest plot of the meta-analysis comparing the overall postoperative femoral head collapse rate.

Seven studies [31–33,35,37,38,43] involving 663 hips reported on the overall postoperative femoral head collapse rate. Heterogeneity was identified across these studies (*P* = 0.006, *I*^*2*^ = 67%), leading to the use of a random-effects model. The analysis revealed a significant difference in the postoperative femoral head collapse rate between the two groups (RR = 0.49, 95% Cl: 0.27 to 0.89, *P* = 0.02) (Fig 6, Table 3).

#### Harris hip score

**Fig 7:**
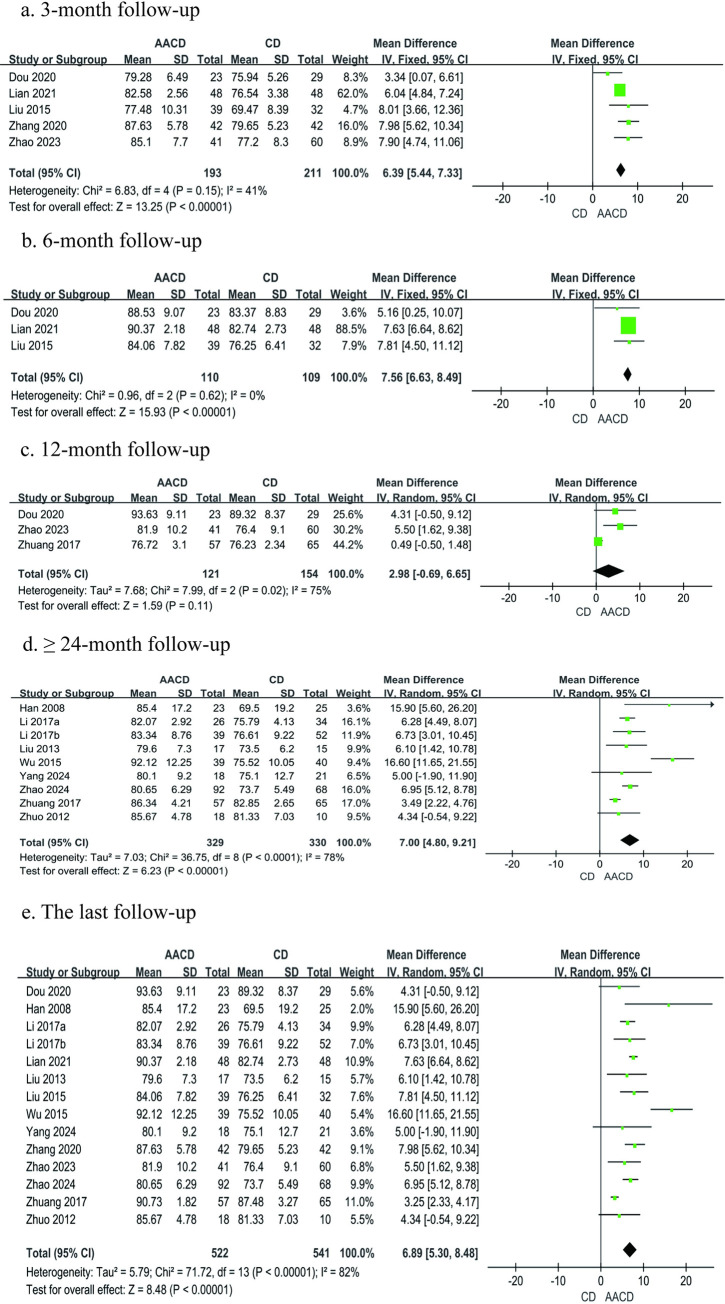
Forest plot of the meta-analysis comparing the Harris hip score: A the duration of follow-up 3 months b the duration of follow-up 6 months c the duration of follow-up 12 months d the duration of follow-up longer than 24 months e the last follow-up.

A total of 14 studies [31–44], comprising 1,063 participants, reported Harris hip scores. Among these, five studies [33–36,40] provided scores at 3 months postoperatively, three studies [34–35,40] at 6 months, three studies [33,35,39] at 12 months, and nine studies [31–32,37–39,41–44] at ≥ 24 months postoperatively. Meta-analysis was conducted based on the last follow-up time points and at different postoperative intervals (3-, 6-, 12-, and ≥ 24-month follow-ups) when such data were available. The results demonstrated a significant difference in Harris hip scores at 3 months, 6 months, ≥ 24 months, and the last follow-up between the two groups (3 months, MD = 6.39, 95% Cl: 5.44 to 7.33, *P* < 0.00001; 6 months, MD = 7.56, 95% Cl: 6.63 to 8.49, *P* < 0.00001; ≥ 24 months, MD = 7.00, 95% Cl: 4.80 to 9.21, *P* < 0.00001; last follow-up, MD = 6.89, 95% Cl: 5.30 to 8.48, *P* < 0.00001). However, no statistically significant difference was observed between the two groups in Harris hip scores at 12 months postoperatively (12 months, MD = 2.98, 95% Cl: -0.69 to 6.65, *P* = 0.11) (Fig 7, Table 3).

#### Overall postoperative complication rate

**Fig 8:**
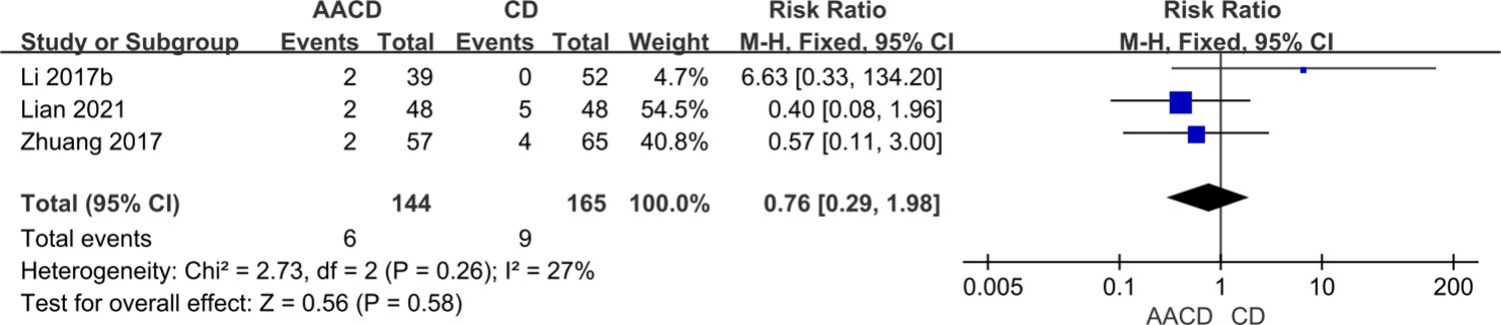
Forest plot of the meta-analysis comparing the overall postoperative complication rate.

Only three studies [34,38,39], involving 309 participants, reported the overall postoperative complication rate. In Lian’s study [34], the treatment group had one case of fat liquefaction and one surgical site infection, compared to two cases of fat liquefaction and three surgical site infections in the control group. Zhuang’s study [39] documented two cases of fat liquefaction or surgical site infection in the treatment group and four in the control group. Li’s study [38] reported two cases of temporary sciatic nerve injury in the treatment group. Given the low heterogeneity (*P* = 0.26, *I*^*2*^ = 27%), a fixed-effects model was applied (Table 2). The result showed no statistically significant difference in overall postoperative complication rates between the two groups (RR = 0.76, 95% Cl: 0.29 to 1.98, P = 0.58) (Fig 8, Table 3).

**Table 2:** Details of reported adverse events from included studies.

Inclusion Studies	Adverse event
Lian 2021 [34]	A:2 (fat liquefaction = 1, surgical site infection = 1); C:5 (fat liquefaction = 2, surgical site infection = 3).
Zhuang 2017 [39]	A:2 (fat liquefaction or surgical site infection = 2); C:4 (fat liquefaction or surgical site infection = 4).
Li 2017 [38]	A:2 (temporary sciatic nerve injury = 2); C:0.

**Note:** A, Arthroscopic-assisted core decompression group; C, Core decompression group.

**Table 3:** Summary data and analyses.

Outcome or subgroup	Statistical methods	Effect estimate	P Value
Operative time	Mean Difference (IV, Random, 95% CI)	31.19 [5.32 to 57.07]	0.02
Intraoperative blood loss	Mean Difference (IV, Random, 95% CI)	2.14 [-7.95 to 12.22]	0.68
Length of hospital stay	Mean Difference (IV, Random, 95% CI)	-0.50 [-2.89 to 1.89]	0.68
Overall postoperative femoral head collapse rate	Risk Ratio (M-H, Random, 95% CI)	0.49 [0.27 to 0.89]	0.02
Harris hip score			
3-month follow-up	Mean Difference (IV, Fixed, 95% CI)	6.39 [5.44 to 7.33]	<0.00001
6-month follow-up	Mean Difference (IV, Fixed, 95% CI)	7.56 [6.63 to 8.49]	<0.00001
12-month follow-up	Mean Difference (IV, Random, 95% CI)	2.98 [-0.69 to 6.65]	0.11
≥ 24-month follow-up	Mean Difference (IV, Random, 95% CI)	7.00 [4.80 to 9.21]	<0.00001
last follow-up	Mean Difference (IV, Random, 95% CI)	6.89 [5.30 to 8.48]	<0.00001
Overall postoperative complication rate	Risk Ratio (M-H, Fixed, 95% CI)	0.76 [0.29 to 1.98]	0.58

### GRADE evaluation

Following the GRADE criteria, we assessed the quality of evidence for various outcomes, including operative time, intraoperative blood loss, length of hospital stay, overall postoperative femoral head collapse rate, Harris hip score, and overall postoperative complication rate. According to S7 Table, the evidence for the overall postoperative complication rate was rated as moderate quality. The evidence for operative time, intraoperative blood loss, overall postoperative femoral head collapse rate, and Harris hip score was classified as low quality, while the length of hospital stay was evaluated as very low quality.

### Sensitivity analysis

To ensure the reliability of the study’s findings, sensitivity analyses were performed for multiple outcome indicators, including operative time, intraoperative blood loss, length of hospital stay, overall postoperative femoral head collapse rate, and Harris hip score. For operative time, the effect size changed after excluding Dou’s study [35] (*I*^*2*^ = 99%, *P* = 0.21), Li’s study [38] (*I*^*2*^ = 99%, *P* = 0.06), and Zhuang’s study [39] (*I*^*2*^ = 100%, *P* = 0.06). For intraoperative blood loss, the effect size shifted following the exclusion of Lian’s study [34] (*I*^*2*^ = 21%, *P* = 0.004), indicating it may be the source of heterogeneity. For the length of hospital stay, the effect size changed directionally after removing Dou’s study [35] (*I*^*2*^ = 91%, *P* = 0.02), suggesting it could be a source of heterogeneity. No other study had a notable impact on the overall effect size. For the overall postoperative femoral head collapse rate, the effect size changed after excluding Zhao’s study [32] (*I*^*2*^ = 0%, *P* = 0.0001), while the Harris hip score effect size shifted after removing Zhuang’s study [39] (*I*^*2*^ = 48%, *P* < 00001). Further details of the sensitivity analyses can be found in S8–S12 Tables.

### Publication bias

We conducted a funnel plot test and Egger’s test for outcomes reported in ten or more studies. The results indicated no significant publication bias. Detailed findings on publication bias are presented in S1 Fig, S13 Table.

## Discussion

Our study found similar outcomes between the two surgical approaches in terms of intraoperative blood loss, length of hospital stay, and overall postoperative complication rate. However, significant differences were noted in operative time, overall postoperative femoral head collapse rate, and Harris hip score.

The longer operative time in the AACD group compared to the CD group aligns with previous studies. Several factors may contribute to this difference. First, AACD requires the establishment of arthroscopic access, which is a time-consuming process. Second, although both procedures are performed by highly experienced surgeons, there are relatively few hospitals offering AACD for ONFH treatment. As a result, AACD remains in the early stages of development and is subject to the learning curve, even for proficient surgical teams. By contrast, CD has been performed for many years, allowing the procedure to be refined, with surgical teams more experienced in working together. Third, some research centers treat additional pathologies during AACD procedures, such as removing hyperplastic synovial tissue or repairing damaged cartilage, which further extends the surgery duration [32,35,38].

Our findings show no significant difference in intraoperative blood loss between the two surgical approaches, consistent with previous literature [33,36]. Excessive blood loss during femoral head surgery increases the risk of perioperative complications. As noted in Lian’s study [34], hip arthroscopy-assisted surgery provides a clear view of the blood vessels, reducing the likelihood of damaging surrounding vessels. However, the data on intraoperative blood loss showed significant heterogeneity, which may be attributed to variations in calculation methods and surgery durations across study centers. Therefore, these results should be interpreted cautiously.

The length of hospital stay for both procedures was also comparable, likely due to the similar nature of the two surgeries. With increased operator familiarity, there should be no significant difference in hospitalization time. However, significant heterogeneity in the data may have been influenced by factors such as differences in rehabilitation outcomes, varying levels of care, and internal hospital management practices [45].

In terms of overall postoperative complication rates, previous studies have reported a higher complication rate for AACD surgery (5.12%) compared to CD surgery (0%) [38]. However, our statistical analysis of the included studies found no significant difference in the overall postoperative complication rates between the two procedures, indicating that both are generally safe. The most common complications associated with both surgeries include infections and postoperative wound issues, most of which are minor and manageable with appropriate postoperative care and rehabilitation. Some experts caution that the anterior approach in hip arthroscopy may pose a risk of femoral artery and nerve damage [33]. To reduce this risk, surgeons often preoperatively mark the course of major nerves and blood vessels and opt for lateral or anterolateral approaches, which can effectively lower the occurrence of complications [37,38].

Interestingly, the overall postoperative femoral head collapse rate was lower in the AACD group compared to the CD group. CD surgery is a widely used hip preservation technique that reduces hydrostatic pressure in the femoral head by creating bone tunnels, which improves bone microcirculation and promotes capillary reconstruction, aiding in bone regeneration and repair [46–48]. However, excessive removal of necrotic tissue during CD surgery has been shown in clinical practice to increase the risk of postoperative femoral head collapse [49–51]. Similarly, studies have found that femoral head collapse after allogeneic fibular grafting often occurs in the anterolateral column sclerotic band, likely due to stress concentration in that area [52,53]. Therefore, preserving the integrity of the anterolateral column and its sclerotic band during surgery is recommended. Zheng’s study confirmed that preserving the blood supply to the lateral column of the femoral head may support implant viability [54], while Zhou’s study concluded that careful debridement, limited to 3/8 to 1/2 of the necrotic area, is more effective in preventing further collapse of the articular surface than complete debridement [55]. The AACD procedure benefits from arthroscopic magnification, enabling more precise identification of the depth and extent of the necrotic area. This precision helps preserve more healthy bone tissue in the femoral head, reducing the likelihood of femoral head collapse caused by excessive removal of necrotic lesions.

The Harris hip score is a comprehensive tool for evaluating hip function, assessing four key dimensions: pain level, deformity, range of motion, and function. Our study showed that AACD offered greater improvements in postoperative Harris hip scores compared to CD. Typically, precise removal of the lesion combined with adequate decompression results in reduced postoperative discomfort and a quicker recovery of joint function. Some studies suggest that early clinical symptoms of ONFH may be associated with hypertrophy of synovial tissue, joint swelling due to free bodies, and cartilage damage [33,38]. In addition to decompressing the femoral head, it is essential not to overlook secondary pathological changes within the hip joint cavity during treatment [56,57]. Hip arthroscopy provides direct visualization of the femoral head’s surface and the internal environment of the joint, allowing for accurate disease assessment. Furthermore, it facilitates the removal of synovial edema, trimming of cartilage, elimination of inflammatory mediators, reduction of pressure within the hip joint, and overall improvement of the joint’s internal environment.

The GRADE system was used to evaluate the quality of the studies included in this meta-analysis. The evidence summary table clearly outlines the process of rating the evidence and the rationale behind the grading, which helps clinicians assess the effectiveness and feasibility of the intervention to make well-informed clinical decisions. In this study, the evidence for the overall postoperative complication rate was rated as moderate quality. Meanwhile, the evidence for operative time, intraoperative blood loss, overall postoperative femoral head collapse rate, and Harris hip score was classified as low quality, and the length of hospital stay was rated as very low quality. This indicates that the methodological quality of the included studies was generally low, with a lack of rigorous experimental design potentially affecting the reliability of the findings. Consequently, some results should be interpreted with caution. The low quality of evidence may be attributed to unclear methodological descriptions in many studies, significant heterogeneity among studies, and potential publication bias. Future research should focus on standardizing experimental design and rigorously implementing protocols to improve the overall quality of evidence. In the clinical management of patients with ONFH, it remains essential to perform a comprehensive assessment of factors such as age, disease duration, the site, extent, and depth of necrosis, to make well-informed clinical decisions.

### Limitations

Although the outcome indicators were evaluated through meta-analysis, this study had several limitations: (1) Most of the included studies were retrospective, with low methodological and evidence quality, which reduced the credibility of the findings; (2) Some outcome indicators were evaluated in a limited number of studies, potentially introducing bias in the results; (3) Variations in disease duration among patients may have contributed to biases in the study outcomes; (4) The complexity and difficulty of the arthroscopic technique led to differences in the surgical approach and experience of operators, which may have impacted the results; (5) Many studies did not thoroughly address the overall postoperative complication rate, meaning the safety of the AACD procedure requires further investigation; (6) Due to limited data in the included literature, subgroup analyses based on factors such as age, etiology, and disease stage could not be performed.

## Conclusion

Based on the available evidence, AACD treatment for ONFH is effective in delaying femoral head collapse, promoting functional recovery, and improving long-term clinical outcomes. However, due to the limitations of this study, further validation through large, high-quality randomized controlled trials is needed to strengthen the credibility of these findings and provide more robust evidence for the treatment of ONFH.

## Supporting information

S1 Table Search strategy. (PDF)

S2 Table PRISMA checklist. (PDF)

S3 Table The data extracted from the studies included in this systematic review that would be needed to replicate this meta-analysis. (PDF)

S4 Table Bias risk assessment results of included retrospective cohort studies. (PDF)

S5 Table Bias risk assessment results of included retrospective case control studies. (PDF)

S6 Table The bias risk for each study in this meta-analysis based on the Cochrane tool. (PDF)

S7 Table GRADE evaluation of evidence quality. (PDF)

S8 Table Sensitivity analysis for operative time. (PDF)

S9 Table Sensitivity analysis for intraoperative blood loss. (PDF)

S10 Table Sensitivity analysis for length of hospital stay. (PDF)

S11 Table Sensitivity analysis for overall postoperative femoral head collapse rate. (PDF)

S12 Table Sensitivity analysis for Harris hip score. (PDF)

S13 Table Publication bias evaluated by egger test. (PDF)

S1 Fig The funnel plot of Harris hip score. (TIF)

S1 File Detailed information of excluded studies. (PDF)

## Abbreviations

AACD: arthroscopic-assisted core decompression
CD: core decompression
CI: confidence interval
GRADE: Grades of Recommendations, Assessment, Development, and Evaluations
ONFH: osteonecrosis of the femoral head
RR: risk ratio
MD: mean difference
