# Supplementary material for: Arthroscopic assisted versus open core decompression for osteonecrosis of the femoral head: A systematic review and meta-analysis
Source: PLoS One. 2024 Nov 15;19(11):e0313265. doi: 10.1371/journal.pone.0313265 (PMC11567543; doi:10.1371/journal.pone.0313265)
Supplement: S1 Table — (PDF) [file pone.0313265.s001.pdf]

Supplementary table1. Search strategy.

Database	Step	Search term
PubMed	#1	((((arthroscopy [Title/Abstract]) OR (arthroscopic [Title/Abstract]) OR (surgery [Title/Abstract])) OR (surgical [Title/Abstract])) OR (operation [Title/Abstract])
	#2	("osteonecrosis of the femoral head" [Mesh]) OR ((ONFH [Title/Abstract])) OR (femur head necrosis [Title/Abstract])
	#3	#1 AND #2
Web of Science	#1	TS=(arthroscopy OR arthroscopic OR surgery OR surgical OR operation)
	#2	TS=(osteonecrosis of the femoral head OR ONFH OR femur head necrosis)
	#3	#1 AND #2
Embase	#1	'arthroscopy'/exp
	#2	'arthroscopic':ab,ti OR 'surgery':ab,ti OR 'surgical':ab,ti OR 'operation':ab,ti
	#3	#1 OR #2
	#4	'osteonecrosis of the femoral head'/exp
	#5	'ONFH':ab,ti' OR 'femur head necrosis':ab,ti
	#6	#4 OR #5
	#7	#3 AND #6
Cochrane Central Register of Controlled Trials	#1	(arthroscopy):ti,ab,kw OR (arthroscopic):ti,ab,kw OR (surgery):ti,ab,kw OR (surgical):ti,ab,kw OR (operation):ti,ab,kw
	#2	MeSH descriptor: [osteonecrosis of the femoral head] explode all trees
	#3	(ONFH):ti,ab,kw OR (femur head necrosis):ti,ab,kw
	#4	#2 OR #3
	#5	#1 AND #4
