# Supplementary material for: Arthroscopic assisted versus open core decompression for osteonecrosis of the femoral head: A systematic review and meta-analysis
Source: PLoS One. 2024 Nov 15;19(11):e0313265. doi: 10.1371/journal.pone.0313265 (PMC11567543; doi:10.1371/journal.pone.0313265)
Supplement: S3 Table — (PDF) [file pone.0313265.s003.pdf]

Supplementary table 4. The data extracted from the studies included in this systematic review that would be needed to replicate this meta-analysis.

Inclusion studies	Region	Study design	Sample	Age (years)	Outcomes						Name of data extractors	Date of data extraction
					Operative time (Mean ± SD)	Intraoperative blood loss (Mean ± SD)	Length of hospital stay (Mean ± SD)	Overall postoperative femoral head collapse rate (Event/Total)	Harris hip score (Mean ± SD)	Overall postoperative complication rate (Event/Total)		
Yang 2024 [31]	China	Retrospective cohort study	A: 18<br>C: 21	A: 39.7 ± 8.5<br>C: 40.8 ± 10.2	NA	NA	NA	A: 2/18<br>C: 6/21	A: 80.1 ± 9.2<br>C: 75.1 ± 12.7	NA	G.M.G. and J.X.	26nd June, 2024
Zhao 2024 [32]	China	Retrospective cohort study	A: 92<br>C: 68	A: 38.21 ± 8.73<br>C: 41.31 ± 10.24	NA	NA	NA	A: 70/92<br>C: 62/68	A: 80.65 ± 6.29<br>C: 73.7 ± 5.49	NA	G.M.G. and J.X.	26nd June, 2024
Zhao 2023 [33]	China	Retrospective cohort study	A: 41<br>C: 60	A: 35.5 ± 9.8<br>C: 37.7 ± 10.5	A: 84.5 ± 15.8<br>C: 52.6 ± 12.5	A: 128.4 ± 26.2<br>C: 119.8 ± 31.7	A: 5.5 ± 1.7<br>C: 5.8 ± 2.1	A: 6/59<br>C: 22/80	A: 81.9 ± 10.2<br>C: 76.4 ± 9.1	NA	G.M.G. and J.X.	26nd June, 2024
Lian 2021 [34]	China	Randomized controlled trial	A: 48<br>C: 48	A: 40.25 ± 5.11<br>C: 40.98 ± 5.83	A: 56.12 ± 10.54<br>C: 79.01 ± 12.37	A: 150.1 ± 17.52<br>C: 161.34 ± 17.1	A: 12.59 ± 2.33<br>C: 15.64 ± 2.68	NA	A: 90.37 ± 2.18<br>C: 82.74 ± 2.73	A: 2/48<br>C: 5/48	G.M.G. and J.X.	26nd June, 2024
Dou 2020 [35]	China	Retrospective cohort study	A: 23<br>C: 29	A: 38.52 ± 7.38<br>C: 36.58 ± 6.91	A: 159.73 ± 22.64<br>C: 36.18 ± 10.82	NA	A: 7.62 ± 2.28<br>C: 4.16 ± 1.08	A: 0/31<br>C: 2/36	A: 93.63 ± 9.11<br>C: 89.32 ± 8.37	NA	G.M.G. and J.X.	26nd June, 2024

Zhang 2020 [36]	China	Randomized controlled trial	A: 42<br>C: 42	A: 41.65 ± 8.42<br>C: 41.28 ± 8.74	A: 79.15 ± 13.81<br>C: 78.43 ± 13.39	A: 155.34 ± 16.87<br>C: 153.06 ± 15.64	A: 14.26 ± 1.26<br>C: 16.33 ± 1.38	NA	A: 87.63 ± 5.78<br>C: 79.65 ± 5.23	NA	G.M.G. and J.X.	26nd June, 2024
Li 2017 [37]	China	Retrospective case control study	A: 26<br>C: 34	A: 37.4 ± 10.3<br>C: 35.2 ± 11.8	NA	NA	NA	A: 6/43<br>C: 14/55	A: 82.07 ± 2.92<br>C: 75.79 ± 4.13	NA	G.M.G. and J.X.	26nd June, 2024
Li 2017 [38]	China	Retrospective cohort study	A: 39<br>C: 52	A: 32.7<br>C: 31.3	A: 73.3 ± 10.6<br>C: 41.5 ± 7.2	NA	NA	A: 6/53<br>C: 16/74	A: 83.34 ± 8.76<br>C: 76.61 ± 9.22	A: 2/39<br>C: 0/52	G.M.G. and J.X.	26nd June, 2024
Zhuang 2017 [39]	China	Randomized controlled trial	A: 57<br>C: 65	NA	A: 80.01 ± 13.72<br>C: 56.21 ± 5.47	A: 161.01 ± 17.06<br>C: 151 ± 21.75	NA	NA	A: 90.73 ± 1.82<br>C: 87.48 ± 3.27	A: 2/57<br>C: 4/65	G.M.G. and J.X.	26nd June, 2024
Liu 2015 [40]	China	Randomized controlled trial	A: 39<br>C: 32	A: 41.36 ± 11.74<br>C: 40.21 ± 9.25	NA	NA	NA	NA	A: 84.06 ± 7.82<br>C: 76.25 ± 6.41	NA	G.M.G. and J.X.	26nd June, 2024
Wu 2015 [41]	China	Retrospective case control study	A: 39<br>C: 40	A: 38.35 ± 10.15<br>C: 37.01 ± 11.25	NA	NA	NA	NA	A: 92.12 ± 12.25<br>C: 75.52 ± 10.05	NA	G.M.G. and J.X.	26nd June, 2024
Liu 2013 [42]	China	Randomized controlled trial	A: 17<br>C: 15	A: 36.5<br>C: 36.5	NA	NA	NA	NA	A: 79.6 ± 7.3<br>C: 73.5 ± 6.2	NA	G.M.G. and J.X.	26nd June, 2024
Zhuo 2012 [43]	China	Retrospective cohort study	A: 18<br>C: 10	A: 32.5<br>C: 30.8	NA	NA	NA	A: 1/21<br>C: 4/12	A: 85.67 ± 4.78	NA	G.M.G.	26nd

									C: 81.33 ± 7.03		and J.X.	June, 2024
Han 2008 [44]	China	Randomized controlled trial	A: 23<br>C: 25	A: 33.6<br>C: 33.6	NA	NA	NA	NA	A: 85.4 ± 17.2<br>C: 69.5 ± 19.2	NA	G.M.G. and J.X.	26nd June, 2024

Note: A, Arthroscopic-assisted core decompression group; C, Core decompression group; NA, not available.
