# Supplementary material for: Arthroscopic assisted versus open core decompression for osteonecrosis of the femoral head: A systematic review and meta-analysis
Source: PLoS One. 2024 Nov 15;19(11):e0313265. doi: 10.1371/journal.pone.0313265 (PMC11567543; doi:10.1371/journal.pone.0313265)
Supplement: S4 Table — (PDF) [file pone.0313265.s004.pdf]

Supplementary table 5. Bias risk assessment results of included retrospective cohort studies.

Study	Selection				Comparability	Outcome			Newcastle-Ottawa Scale
	Q1	Q2	Q3	Q4	Q5	Q6	Q7	Q8	Overall score
Yang 2024 [31]	1	1	1	1	1	1	1	1	8
Zhao 2024 [32]	1	1	1	1	1	1	1	1	8
Zhao 2023 [33]	1	1	1	1	1	1	0	1	7
Dou 2020 [35]	1	1	1	1	1	1	0	1	7
Li 2017 [38]	1	1	1	1	1	1	1	1	8
Zhuo 2012 [43]	1	1	1	1	1	1	1	1	8

Notes: Q1. Representativeness of the exposed group; Q2. Representativeness of the non-exposed group; Q3. Identification of exposure factors; Q4. Confirmation of no outcome indicators to be observed at the beginning of the study; Q5. Comparability on the basis of the design or analysis; Q6. Assessment of outcome indicators; Q7. Adequate follow-up duration; Q8. Completeness of follow-up between the exposed and unexposed groups.
