# Supplementary material for: Arthroscopic assisted versus open core decompression for osteonecrosis of the femoral head: A systematic review and meta-analysis
Source: PLoS One. 2024 Nov 15;19(11):e0313265. doi: 10.1371/journal.pone.0313265 (PMC11567543; doi:10.1371/journal.pone.0313265)
Supplement: S5 Table — (PDF) [file pone.0313265.s005.pdf]

Supplementary table 6. Bias risk assessment results of included retrospective case control studies.

Study	Selection				Comparability	Outcome			Newcastle-Ottawa Scale
	Q1	Q2	Q3	Q4	Q5	Q6	Q7	Q8	Overall score
Li 2017 [37]	1	1	1	1	1	1	1	1	8
Wu 2015 [41]	1	1	1	1	1	1	1	1	8

Notes: Q1. Adequate definition of the case; Q2. Representativeness of the cases; Q3. Selection of controls; Q4. Definition of controls; Q5. Control for important factor; Q6. Ascertainment of exposure; Q7. Same method of ascertainment for cases and controls; Q8. Nonresponse rate.
