# Supplementary material for: Arthroscopic assisted versus open core decompression for osteonecrosis of the femoral head: A systematic review and meta-analysis
Source: PLoS One. 2024 Nov 15;19(11):e0313265. doi: 10.1371/journal.pone.0313265 (PMC11567543; doi:10.1371/journal.pone.0313265)
Supplement: S6 Table — (PDF) [file pone.0313265.s006.pdf]

Supplementary table 7. The bias risk for each study in this meta-analysis based on the Cochrane tool.

Inclusion studies	Random sequence generation	Allocation concealment	Blinding of participants and personnel	Blinding of outcome assessment	Incomplete outcome data	Selective reporting	Other biases
Lian 2021 [34]	Low	Low	Unclear	Unclear	Unclear	Low	Low
Zhang 2020 [36]	Unclear	High	High	Unclear	Unclear	High	Low
Zhuang 2017 [39]	Unclear	High	Unclear	Unclear	Unclear	Low	Low
Liu 2015 [40]	Unclear	High	High	Unclear	Unclear	High	Low
Liu 2013 [42]	Low	Unclear	Unclear	Unclear	Low	High	Low
Han 2008 [44]	Low	Unclear	Unclear	Unclear	Low	High	Low
