# Supplementary material for: Arthroscopic assisted versus open core decompression for osteonecrosis of the femoral head: A systematic review and meta-analysis
Source: PLoS One. 2024 Nov 15;19(11):e0313265. doi: 10.1371/journal.pone.0313265 (PMC11567543; doi:10.1371/journal.pone.0313265)
Supplement: S7 Table — (PDF) [file pone.0313265.s007.pdf]

Supplementary table 8. GRADE evaluation of evidence quality.

Outcome, number of sties (number of participants)	Limitation in study design [1]	Risk of bias [2]	Inconsistency of results [3]	Indirectness [4]	Imprecision [5]	Publication bias [6]	Overall quality of evidence
Operative time<br>6 Studies (584)	-1	0	-1	0	0	0	Low
Intraoperative blood loss<br>4 Studies (403)	-1	0	-1	0	0	0	Low
Length of hospital stay<br>4 Studies (333)	-1	0	-1	0	-1	0	Very low
Overall postoperative femoral head collapse rate<br>7 Studies (663)	-1	0	-1	0	0	0	Low
Harris hip score<br>14 Studies (1063)	-1	0	-1	0	0	0	Low
Overall postoperative complication rate<br>3 Studies (309)	-1	0	0	0	0	0	Moderate

3 Inconsistency of results.The extent of overlap of confidence intervals and I^2 test			
Variable	Confidence intervals overlapping	Test of heterogeneity	I^2
Operative time	Yes	P < 0.00001 (significant)	99% (substantial)
Intraoperative blood loss	Yes	P = 0.0001 (significant)	85% (substantial)
Length of hospital stay	Yes	P < 0.00001 (significant)	97% (substantial)
Overall postoperative femoral head collapse rate	Yes	P = 0.006 (significant)	67% (substantial)
Harris hip score	Yes	P < 0.00001 (significant)	82% (substantial)
Overall postoperative complication rate	Yes	P = 0.26 (non-significant)	27% (moderate)

6 Publication bias. Summary of funding according to the outcome variables				
Variable	Number of studies that reported no funding or no influence	Number of studies that unreported funding	Number of studies that reported any type of funding	Total
Operative time	0	4	2	6
Intraoperative blood loss	0	2	2	4

Length of hospital stay	0	3	1	4
Overall postoperative femoral head collapse rate	1	4	2	7
Harris hip score	1	10	3	14
Overall postoperative complication rate	0	2	1	3

6 Publication bias. Summary of conflict of interest according to the outcome variables			
Variable	Number of studies declaring no conflict of interest	Number of studies unreported conflict of interest	Total
Operative time	1	5	6
Intraoperative blood loss	1	3	4
Length of hospital stay	1	3	4
Overall postoperative femoral head collapse rate	4	3	7
Harris hip score	5	9	14
Overall postoperative complication rate	0	3	3

Rate down if: [1] Observational study.

[2] > 25 % of the participants from studies with a high risk of bias.

[3] Heterogeneity was based on the extent of overlap of confidence intervals and I^2 test (>50 %);

[4] > 25 % of studies had a poor representation of outcome (when definitions of the outcome varied) or population (non-general population);

[5] Fewer than 300 participants pooled binary data or fewer than 400 participants pooled continuous data.

[6] Publication bias was based on the funnel plot and Egger's test (*Unclear for outcomes with less than 10 studies included), the sample size of the included studies, sponsorship and/or conflict of interest report.
