# Supplementary material for: Arthroscopic assisted versus open core decompression for osteonecrosis of the femoral head: A systematic review and meta-analysis
Source: PLoS One. 2024 Nov 15;19(11):e0313265. doi: 10.1371/journal.pone.0313265 (PMC11567543; doi:10.1371/journal.pone.0313265)
Supplement: S8 Table — (PDF) [file pone.0313265.s008.pdf]

Supplementary table 9. Seneitivity analysis for operative time.

Eliminated study	Heterogeneity		Effect Model	MD	95% CI	P Value
	P Value	I^2 (%)				
None	<0.00001	99	Random	31.19	5.32 to 57.07	0.02
Zhao 2023 [33]	<0.00001	100	Random	31.11	-0.24 to 62.47	0.05
Lian 2021 [34]	<0.00001	99	Random	41.91	18.57 to 65.26	0.0004
Dou 2020 [35]	<0.00001	99	Random	13.09	-7.49 to 33.68	0.21
Zhang 2020 [36]	<0.00001	99	Random	37.32	7.37 to 67.27	0.01
Li 2017 [38]	<0.00001	99	Random	31.16	-1.64 to 63.95	0.06
Zhuang 2017 [39]	<0.00001	100	Random	32.78	-1.02 to 66.58	0.06
