# Supplementary material for: Arthroscopic assisted versus open core decompression for osteonecrosis of the femoral head: A systematic review and meta-analysis
Source: PLoS One. 2024 Nov 15;19(11):e0313265. doi: 10.1371/journal.pone.0313265 (PMC11567543; doi:10.1371/journal.pone.0313265)
Supplement: S9 Table — (PDF) [file pone.0313265.s009.pdf]

Supplementary table 10. Seneitivity analysis for intraoperative blood loss.

Eliminated study	Heterogeneity		Effect Model	MD	95% CI	P Value
	P Value	I^2 (%)				
None	0.0001	85	Random	2.14	-7.95 to 12.22	0.68
Zhao 2023 [33]	<0.0001	89	Random	0.35	-11.84 to 12.54	0.95
Lian 2021 [34]	0.28	21	Fix	6.56	2.06 to 11.06	0.004
Zhang 2020 [36]	<0.0001	90	Random	2.22	-12.69 to 17.13	0.77
Zhuang 2017 [39]	0.003	83	Random	-0.68	-12.03 to 10.66	0.91

1
