# Supplementary material for: Arthroscopic assisted versus open core decompression for osteonecrosis of the femoral head: A systematic review and meta-analysis
Source: PLoS One. 2024 Nov 15;19(11):e0313265. doi: 10.1371/journal.pone.0313265 (PMC11567543; doi:10.1371/journal.pone.0313265)
Supplement: S10 Table — (PDF) [file pone.0313265.s010.pdf]

Supplementary table 11. Seneitivity analysis for length of hospital stay.

Eliminated study	Heterogeneity		Effect Model	MD	95% CI	P Value
	P Value	I^2 (%)				
None	<0.00001	97	Random	-0.50	-2.89 to 1.89	0.68
Zhao 2023 [33]	<0.00001	98	Random	-0.56	-4.08 to 2.95	0.75
Lian 2021 [34]	<0.00001	98	Random	0.34	-2.55 to 3.22	0.82
Dou 2020 [35]	<0.0001	91	Random	-1.78	-3.23 to -0.33	0.02
Zhang 2020 [36]	<0.00001	98	Random	0.03	-3.32 to 3.39	0.98
