# Supplementary material for: Arthroscopic assisted versus open core decompression for osteonecrosis of the femoral head: A systematic review and meta-analysis
Source: PLoS One. 2024 Nov 15;19(11):e0313265. doi: 10.1371/journal.pone.0313265 (PMC11567543; doi:10.1371/journal.pone.0313265)
Supplement: S11 Table — (PDF) [file pone.0313265.s011.pdf]

Supplementary table 12. Seneitivity analysis for overall postoperative femoral head collapse rate.

Eliminated study	Heterogeneity		Effect Model	RR	95% CI	P Value
	P Value	I^2 (%)				
None	0.006	67	Random	0.49	0.27 to 0.89	0.02
Yang 2024 [31]	0.007	69	Random	0.50	0.27 to 0.94	0.03
Zhao 2024 [32]	0.86	0	Fix	0.42	0.27 to 0.66	0.0001
Zhao 2023 [33]	0.06	53	Random	0.55	0.31 to 0.97	0.04
Dou 2020 [35]	0.005	70	Random	0.50	0.27 to 0.92	0.03
Li 2017 [37]	0.006	70	Random	0.46	0.23 to 0.95	0.04
Li 2017 [38]	0.007	69	Random	0.47	0.23 to 0.95	0.04
Zhuo 2012 [43]	0.02	62	Random	0.54	0.31 to 0.95	0.03
