# Supplementary material for: Arthroscopic assisted versus open core decompression for osteonecrosis of the femoral head: A systematic review and meta-analysis
Source: PLoS One. 2024 Nov 15;19(11):e0313265. doi: 10.1371/journal.pone.0313265 (PMC11567543; doi:10.1371/journal.pone.0313265)
Supplement: S12 Table — (PDF) [file pone.0313265.s012.pdf]

Supplementary table 13. Seneitivity analysis for Harris hip score.

Eliminated study	Heterogeneity		Effect Model	MD	95% CI	P Value
	P Value	I^2 (%)				
None	<0.00001	82	Random	6.89	5.30 to 8.48	<0.00001
Yang 2024 [31]	<0.00001	83	Random	6.96	5.33 to 8.60	<0.00001
Zhao 2024 [32]	<0.00001	83	Random	6.91	5.15 to 8.67	<0.00001
Zhao 2023 [33]	<0.00001	83	Random	7.00	5.32 to 8.67	<0.00001
Lian 2021 [34]	<0.00001	78	Random	6.83	5.07 to 8.58	<0.00001
Dou 2020 [35]	<0.00001	83	Random	7.05	5.39 to 8.70	<0.00001
Zhang 2020 [36]	<0.00001	83	Random	6.79	5.09 to 8.49	<0.00001
Li 2017 [37]	<0.00001	83	Random	6.99	5.21 to 8.76	<0.00001
Li 2017 [38]	<0.00001	83	Random	6.91	5.23 to 8.59	<0.00001
Zhuang 2017 [39]	0.03	48	Fix	7.31	6.64 to 7.89	<0.00001
Liu 2015 [40]	<0.00001	83	Random	6.82	5.14 to 8.50	<0.00001
Wu 2015 [41]	<0.00001	78	Random	6.31	4.85 to 7.77	<0.00001
Liu 2013 [42]	<0.00001	83	Random	6.94	5.28 to 8.60	<0.00001
Zhuo 2012 [43]	<0.00001	83	Random	7.04	5.39 to 8.70	<0.00001

Han 2008 [44]	<0.00001	82	Random	6.70	5.12 to 8.28	<0.00001
