# Supplementary material for: Arthroscopic assisted versus open core decompression for osteonecrosis of the femoral head: A systematic review and meta-analysis
Source: PLoS One. 2024 Nov 15;19(11):e0313265. doi: 10.1371/journal.pone.0313265 (PMC11567543; doi:10.1371/journal.pone.0313265)
Supplement: S13 Table — (PDF) [file pone.0313265.s013.pdf]

Supplementary table 14. Publication bias evaluated by egger test.

Outcomes	Number of trails	Egger's test (P value)
Harris hip score	14 [31-44]	0.203
