# Supplementary material for: Arthroscopic assisted versus open core decompression for osteonecrosis of the femoral head: A systematic review and meta-analysis
Source: PLoS One. 2024 Nov 15;19(11):e0313265. doi: 10.1371/journal.pone.0313265 (PMC11567543; doi:10.1371/journal.pone.0313265)
Supplement: S1 File — (PDF) [file pone.0313265.s015.pdf]

In total, 156 records were identified after removing duplicates, among which 57 records were removed for being reviews [1-57], 43 records were removed as being studies with other interventions [58-100], 15 records were removed for being animal experiments [101-115], 9 records were removed for being lach of relative outcomes [116-124], 8 were removed for being wrong interventions [125-132], 5 were removed for being wrong statistical methods [133-137], and 5 were removed for being incomplete data [138-142], leaving 14 studies to be included in the final analysis [143-156].

References

1. Wu, Y. D., Yu, K. K., Liu, C. B., Liu, Y. J., 2022. Choice of indications and contraindications for hip arthroscopy. Orthopedic Journal of China, 30(05):431-435.

2. Chen, Y. H., Luo, C., 2020. Progress in the study of the treatment of developmental hip dysplasia in children aged 6 months to 4 years. Journal of Modern Medicine & Health, 36(23):3825-3828.

3. Li, C. C., Ye, G. Z., Liu, W. G., Chen, G. C., Huang, Z. Q., Xue, Z. P., et al., 2020. Current status of application of surgery and western medicine to treatment of osteonecrosis of the femoral head. The Journal of Traditional Chinese Orthopedics and Traumatology, 32(01):43-46+55.

4. Hua, S. L., Wei, W., Long, Z. X., Wang, L. Y., Pan, S. C., 2019. Progress in the study of core decompression of ischaemic necrosis of the femoral head and its related treatment. Journal of Youjiang Medical University for Nationalities, 41(01):103-106.

5. Chen, X. W., Wang, Y. W., 2018. Advances in hip arthroscopy techniques. World Latest Medicine Information, 18(72):143-144.

6. Li, T. X., Chen, Z. W., Wang, R. T., Huang, J. H., Liu, D. B., He, H. J., et al., 2018. Advances in the treatment of epiphyseal necrosis of the femoral head in children. Chinese Journal of Bone and Joint Injury, 33(02):221-222.

7. Hong, Z. N., He, W., Wei, Q. S., Tang, H. Y., Yang, F., Guan, T. A., 2017. Current physiotherapy for osteonecrosis of the femoral head. Orthopedic Journal of China, 25(23):2160-2164.

8. Ling, G. H., Yao, L., Ou, Z. H., Wen, L. C., Wang, G. X., Li, Y. B., et al., 2017. Research progress of early and middle stage hip preservation therapy for adult femoral head necrosis. Rheumatism and Arthritis, 6(04):60-64.

9. Liu, J., Hao, Y. Q., Wang, Y. G., Zhang, B. G., Liu, Z. Y., 2017. Progress in the study of head preserving treatment of femoral head necrosis. Hunan Journal of Traditional Chinese Medicine, 33(01):181-184.

10. Pan, X. W., Xie, X. W., Huang, J., Lyu, L. T., Jiang, G. P., Bai, B. H., 2016. Progress of research on hip preservation therapy for femoral head necrosis. Chinese Journal of Traditional Medical Traumatology & Orthopedics, 24(12):74-78.

11. Wang, P., Wang, Y. W., Zhang, G. L., 2016. Progress in clinical surgical treatment of ischaemic necrosis of the femoral head in adults. World Latest Medicine Information, 16(63):34-35.

12. Xing, G. Y., Liu, S. T., Wu, K., Liu, Y., 2015. Perspectives of extracorporeal shock wave and its arthroscopic combination therapy in the treatment of osteoarthritis and osteonecrosis of the bone. Chinese Journal of the Frontiers of Medical Science (Electronic Version), 7(11):1-5.

13. Tian, Q. S., Zhou, Y., Li, X. Z., 2014. Advances in the diagnosis and treatment of early necrosis of the femoral head. The Journal of Practical Medicine, 30(18):2869-2871.

14. Wang, J., Yu, S., 2013. Advances in the treatment of ischaemic necrosis of the femoral head in children. Journal of zunyi medical university, 36(05):495-499.

15. Zhang, Z. L., Lu, Y., 2013. Current status of treatment of paediatric developmental hip dislocation. Xinjiang medicine, 43(05):1-5.

16. Wang, R., Tang, J., Wang, W. J., 2013. Research progress of ischaemic necrosis of femoral head. World Chinese Medicine, 8(05):586-588.

17. Teng, J. Z., Yan, S. H., Miao, Z. X., Su, B., 2013. Progress of minimally invasive hip-preserving treatment for ischaemic necrosis of the femoral head in adults. Minimally Invasive Medicine, 8(02):202-204.

18. Jiang, W., Shang, X. F., 2013. Progress of head preservation therapy for femoral head necrosis. Medical Review, 19(01):100-104.

19. Sun, K., Yin, D., 2010. Progress of clinical application of arthroscopy in hip joint diseases[J]. China Clinical New Medicine, 3(12):1250-1253.

20. Wang, J. Y., Li, G., 2010. Progress of core decompression in the treatment of femoral head necrosis. Medical Review, 16(17):2643-2646.

21. Li, X., Dong, J. Y., Hao, L. B., 2010. Early diagnosis of femoral head necrosis. China Medical Journal, 45(02):20-23.

22. Huang, Z., Yin, H., Xie, J., 2009. Research progress of hip arthroscopic surgery. Chinese Journal of Orthopaedic Surgery, 17(09):684-687.

23. Li, J., 2009. The diagnosis and treatment of hip joint disorders. Journal of the Fourth Military Medical University, 30(08):673-675.

24. Huang, Z., Yin, H., Xie, J., 2009. Hip arthroscopy. International Journal of Orthopaedics, 30(01):42-44.

25. Wang, Q., Jin, Z. Y., Chen, W. H., 2008. Current status of clinical application of hip arthroscopy technology. Chinese Journal of Bone and Joint Injury, (10):877-879.

26. Li, R. Q., Zhang, G. P., Ren, L. Z., Zhao, F., Gao, H. Y., Wang, W., 2008. Research progress in the treatment of ischaemic necrosis of femoral head. Modern Journal of Integrative Medicine, (26):4208-4209.

27. Hu, Y. G., 2008. Application of lumboendoscopy in paediatric orthopaedic surgery. Journal of Clinical Paediatric Surgery, (04):58-59.

28. Wu, B. Q., Zhang, Z. Q., Wei, X. C., 2007. Current status of clinical application of hip arthroscopy. Journal of Practical Orthopaedics, (07):407-410.

29. Liu, Y., Xiang, Y., 2005. Advances in hip arthroscopic surgery. Chinese Journal of Orthopaedic Surgery, (21):61-63.

30. Ding, X. J., Cheng, M. Z., Zhang, Y. J., Zhang, Y., 2023. Progress in the clinical application of minimally invasive needlescopy for the treatment of joint-like diseases. Rheumatism and Arthritis, 12(4):67-70.

31. Wang, X. S., 2022. Hip arthroscopy in China with unlimited potential. Journal of Orthopaedic Clinics and Research, 7(4):195-197.

32. Zhou, L., Kong, Q., Zhou, Z. Y., Duan, G. Q., 2022. Research progress of femoroacetabular impingement syndrome. China Clinical Practical Medicine, 13(3):78-80.

33. Zeng, X. H., Liang, B. W., 2021. A new strategy for hip preservation treatment of femoral head necrosis. Chinese Tissue Engineering Research, 25(3):431-437.

34. Zhao, Y., Shen, J. R., Zhang, C., Xue, Z. P., Chen, W. H., 2020. Current status of hip-preserving surgical treatment for osteonecrosis of the femoral head. Chinese Orthopaedics, 32(1):47-5055.

35. Jia, G. Q., Sun, J., 2019. Progress of research on diagnosis and treatment of slipped capital

femoral epiphysis in children. International Journal of Paediatrics, 46(6):449-452.

36. Liu, Y. J., Li, Z. L., Wang, Z. G., Yuan, X. C., Wang, Y., 2002. Application of arthroscopy in the diagnosis and treatment of hip joint disorders. Chinese Journal of Surgery, 40(12):912-915.

37. Han, Y. S., 2002. Surgical treatment of chronic arthritis. China Clinical Rehabilitation, 6(5):629-629641.

38. Guo, D. L., 2020. Application of enhanced MRI examination in the diagnosis of early femoral head necrosis. Clinical Medical Engineering, 27(4):395-396.

39. Rao, X. M., 2017. Progress of the application of the method of tonifying the kidney and activating blood in the treatment of various systemic diseases. Hunan Journal of Traditional Chinese Medicine, 33(8):210-212.

40. Qu, G. H., 2012. Diagnostic value of MRI for bone and joint lesions. Chinese and foreign medical treatment, 31(32):162,164.

41. Bao, Y. S., Wang, J. H., Li, X. J., Zhang, Q., Liu, P., 2009. Clinical study of minimally invasive decompression bone grafting with modified medullary core in the treatment of femoral head necrosis. Contemporary Medicine, 15(6):72-73.

42. Liu, Y. J., Li, Z. L., 2006. Arthropathy and arthroscopy. Drugs and People, (6):70-71.Crofts, H., McConkey, M., Lodhia, P., 2023. Pediatric Hip Arthroscopy: a Review of Indications and Treatment Outcomes. Current reviews in musculoskeletal medicine, 16(7), 284-294.

43. Ng, M. K., Kobryn, A., Golub, I. J., Piuzzi, N. S., Wong, C. H. J., Jones, L., et al., 2023. Increasing trend toward joint-preserving procedures for hip osteonecrosis in the United States from 2010 to 2019. Arthroplasty (London, England), 5(1), 23.

44. Shahpari, O., Mortazavi, J., Ebrahimzadeh, M. H., Bagheri, F., Mousavian, A., 2022. Role of Hip Arthroscopy in the Treatment of Avascular Necrosis of the Hip: A Systematic Review. The archives of bone and joint surgery, 10(6), 480-489.

45. Kunze, K. N., Sullivan, S. W., Nwachukwu, B. U., 2022. Updates on Management of Avascular Necrosis Using Hip Arthroscopy for Core Decompression. Frontiers in surgery, 9, 662722.

46. Zhao, F. Y., Dong, H. M., Huang, H. J., Gao, G. Y., Wu, R. Q., Shi, Y. Y., et al., 2021. The Application of Arthroscopic Techniques in the Diagnosis and Treatment of Hip-Related Conditions in China. Orthopaedic surgery, 13(6), 1697-1706.

47. Crofts, H., McConkey, M., Lodhia, P., 2023. Pediatric Hip Arthroscopy: a Review of Indications and Treatment Outcomes. Current reviews in musculoskeletal medicine, 16(7), 284–294.

48. Tee, Q. X., Nambiar, M., Mahendru, G., Singh, P., 2022. Cooled radiofrequency ablation for pain related to Perthes' disease: a novel application. BMJ case reports, 15(3), e247916.

49. Serong, S., Haubold, J., Theysohn, J., Landgraeber, S., 2020. Arthroscopic assessment of concomitant intraarticular pathologies in patients with osteonecrosis of the femoral head. Journal of hip preservation surgery, 7(3), 458-465.

50. Lim, C., Cho, T. J., Shin, C. H., Choi, I. H., Yoo, W. J., 2020. Functional Outcomes of Hip Arthroscopy for Pediatric and Adolescent Hip Disorders. Clinics in orthopedic surgery, 12(1), 94-99.

51. Theopold, J., Armonies, S., Pieroh, P., Hepp, P., Roth, A., 2020. Nontraumatic avascular necrosis of the femoral head : Arthroscopic and navigation-supported core decompression. Atraumatische avaskuläre Femurkopfnekrose : Arthroskopisch und navigationsgestützte Hüftkopfanbohrung. Operative Orthopadie und Traumatologie, 32(2), 107-115.

52. Papavasiliou, A. V., Triantafyllopoulos, I., Paxinos, O., Tsoukas, D., Kostantoulakis, C., 2018. The role of cell therapies and hip arthroscopy in the management of osteonecrosis: an update. Journal of hip preservation surgery, 5(3), 202-208.

53. Polesello, G. C., Queiroz, M. C., de Figueiredo, M. J. P. S. S., Braga, S. R., Ricioli, W., Jr, Akkari, M., 2017. Medial hip arthroscopy portals: a novel approach for hip pathologies. Is it feasible?. Hip international : the journal of clinical and experimental research on hip pathology and therapy, 27(3), e3-e5.

54. Levy, D. M., Hellman, M. D., Haughom, B., Stover, M. D., Nho, S. J., 2015. Techniques and Results for Open Hip Preservation. Frontiers in surgery, 2, 64.

55. Papavasiliou, A., Yercan, H. S., Koukoulias, N., 2014. The role of hip arthroscopy in the management of osteonecrosis. Journal of hip preservation surgery, 1(2), 56-61.

56. Leunig, M., Ganz, R., 2014. The evolution and concepts of joint-preserving surgery of the hip. The bone & joint journal, 96-B(1), 5-18.

57. Hellman, M. D., Riff, A. J., Haughom, B. D., Patel, R., Stover, M. D., Nho, S. J., 2013. Operative treatment of FAI: open hip preservation surgery. Current reviews in musculoskeletal

medicine, 6(3), 258-263.
58. Wang, Y. T., An, B. J., Wang, L., Song, L. K., Zhao, B., Li, C. B., et al., 2022. Arthroscopic debridement and drilling decompression combined with extracorporeal shockwave therapy for early-stage femoral head necrosis. Chinese Journal of Orthopaedic Surgery, 30(05):421-425.
59. Cheng, S., Ye, X. Y., Tang, L. X., Wang, H. L., Ma, Y., Zhao, Y. G., 2020. Treatment of early femoral head necrosis with Arthroscopic Monitoring of core decompression and bone grafting combined with Nanomimetic Bone Scaffold. South China Journal of Defence Medicine, 34(10):695-698.
60. Huang, H., Xu, X. F., 2020. A case of arthroscopic surgical biopsy in rapidly destructive hip arthritis. Orthopaedics, 11(03):259-261.
61. Feng, C., Wan, S. Q., Lyu, X. M., Guo, Y., 2020. Traction table assisted arthroscopic reduction of developmental hip dislocation in children. Chinese Journal of Orthopaedic Surgery, 28(09):841-845.
62. Song, M. G., Li, J. T., Xue, Z. P., Sun, J. G., Huang, Z. Q., Zhao, Y., et al., 2020. Analysis of clinical effects of hip-preserving treatment of osteonecrosis of the femoral head. Chinese orthopedics, 32(01):36-39+42.
63. Zhang, M. J., Geng, H. L., Liu, H. F., Peng, L. Q., Xu, J., Ou, Y. K., et al., 2019. Diagnosis and arthroscopic treatment for intra-articular injuries following traumatic hip dislocation. Chinese Journal of Bone and Joint Surgery, 12(05):351-355.
64. Zhong, H. H., Yang, J., Liu, Y., 2018. Study on arthroscopic-assisted core decompression combined with tantalum rod placement in the treatment of early femoral head necrosis. Medical Information, 31(20):186-188.
65. Deng, X. T., Liu, J. C., Yang, S. C., Wang, X. Y., Li, Z., 2018. Application of arthroscopic surgery combined with direct anterior approach in hip diseases. Chinese Journal of Repair and Reconstructive Surgery, 32(09):1167-1171.
66. Zhu, M., Zhao, S. X., 2018. Clinical research on arthroscopic surgery for hip joint diseases. Chinese and foreign medical treatment, 37(09):27-29.
67. Guo, H. S., Tian, Y. J., Liu, G., Ang, L., Zhou, Z. G., Liu, H. Z., 2018. Arthroscopy-guided core decompression and bone grafting combined with selective arterial infusion for treatment of early stage avascular necrosis of femoral head. China Bone Injury, 31(01):56-61.

68. Liang, H., 2017. Study on the efficacy of vivifying blood circulation and collaterals in the treatment of non-traumatic ischaemic necrosis of the femoral head. Chinese and Foreign Medicine, 36(33):183-185.

69. Xu, H. F., Huang, L. Y., Lei, W., Sha, J., Li, C., Xu, C., et al., 2017. A Long-term Follow-up of Arthroscopic-assisted Surgery for Developmental Dislocation of Hip in Infants. Chinese Journal of Minimally Invasive Surgery, 17(11):1030-1034.

70. Yang, X. M., Shi, W., Du, Y. K., Zhang, L., Hu, C. B., Meng, X. Y., 2016. Arthroscopy assisted lesion clearance and bone graft, titanium rod support for earlyd bone graft, titanium rod support for earlystage osteonecrosis of the femoral heastage osteonecrosis of the femoral head: survival analysis of the femoral headd: survival analysis of the femoral head. Chinese Journal of Bone and Joint Surgery, 9(05):394-397.

71. Wang, Z. Q., Ye, Y. J., Sun, G. J., Peng, X., Li, Q. S., Yang, S. J., et al., 2016. Analysis of the efficacy of small-diameter multi-orifice channel medullary core decompression combined with hip arthroscopic clean-up in the treatment of early ischaemic necrosis of the femoral head. Modern Medicine and Health, 32(17):2700-2702.

72. Yue, Y. H., Gong, Q. G., Zhou, G., Xiang, K., Shui, G. J., Gong, W. J., et al., 2016. Clinical study of arthroscopic treatment of hip joint diseases. Modern Biomedical Progress, 16(18):3540-3543+3472.

73. Zhao, Y. B., Cheng, Y. D., Du, Y. S., Yue, M. S., Zhang, C. X., Deng, Z. G., et al., 2015. Clinical efficacy of DBM implantation combined with traditional Chinese medicine in the treatment of femoral head necrosis after microscopic lesion removal. Hebei Medicine, 37(22):3398-3400.

74. Tian, Q., Li, X. S., Zhou, S. H., Gao, M. X., 2015. Mid-term efficacy of arthroscopy-assisted surgery for developmental hip dislocation in children. Chinese Journal of Bone and Joint, 4(07):557-560.

75. Shen, J. T., Wang, S. Z., Jiang, Q. L., Zhao, X. L., 2015. Analysis of the application effect of arthroscopy in the diagnosis and treatment of hip joint diseases. China Medical Guide, 13(18):184-185.

76. Chen, X., Yu, J. W., Zhao, Y. L., Ji, D., Li, D. R., Wang, B., et al., 2013. Arthroscopic-assisted medullary decompression combined with compression bone grafting in the treatment of femoral

head necrosis in 17 cases. China Practical Medicine, 8(29):103-104.

77. Chen, F., 2013. Arthroscopic drilling and decompression of autologous cancellous bone combined with autologous bone marrow stromal cell transplantation for the treatment of femoral head necrosis. Nursing Research, 27(10):907-908.

78. Cui, D. P., Zhao, D. W., 2012. Computer navigation-assisted autologous stem cell replantation for early femoral head necrosis. Chinese Tissue Engineering Research, 16(45):8453-8459.

79. Feng, Z. B., Zhang, S. L., Wang, G. H., Wei, R. Z., 2012. Arthroscopic compression bone grafting in the treatment of ischaemic necrosis of femoral head in 28 cases. Guangxi Medicine, 34(10):1367-1368.

80. Li, K. Y., Huang, Y. X., Li, Y. L., Ma, C. P., Yi, W. H., Ou, D. J., 2012. Treatment with BAM artificial bone and autogenous marrow cells implantation after arthroscopic core decompression for ischemic necrosis of femoral head. Journal of Hainan Medical College, 18(11):1557-1559+1562.

81. Liu, J. K., Nie, G. R., Liu, X. X., Guo, J. C., Shi, S. F., 2012. Arthroscopic cleaning, medullary core decompression, focal dead bone scraping and bone grafting for the treatment of ischaemic necrosis of femoral head. China Medical Guide, 10(16):181-182.

82. Liang, J., Liu, A. M., 2012. Observation on the efficacy of hip arthroscopic cleaning and core decompression plus autologous bone graft in the treatment of early femoral head necrosis. Journal of Trauma Surgery, 14(03):268+280.

83. Zhu, M., Yu, S., Hu, Y. G., Yang, X. H., 2011. Report of 39 cases of early ischaemic necrosis of femoral head in children treated by arthroscopic surgery. Guizhou Medicine, 35(12):1102-1103.

84. Sun, K., Yin, D., Liang, B., Tang, G. S., 2011. Clinical efficacy analysis of arthroscopic diagnosis and treatment of hip joint diseases. Chinese Journal of Endoscopy, 17(09):944-947.

85. Wang, Z. G., Wei, M., Liu, Y. J., Li, Z. L., Zhu, J. L., Liu, Y., et al., 2011. Eccentric drill decompression followed by injectable calcium sulfate implantation for early femoral head necrosis. Journal of the College of Continuing Military Medicine, 32(08):788-790.

86. Geng, X. L., Yu, M., 2008. Clinical analysis of early treatment of ischaemic necrosis of femoral head. China Medical Guide, 6(24):89.

87. Chen, W. H., Xu, Z. J., Zhang, C., Liu, D. B., Yin, T., Xie, B., 2008. Clinical research on treating hip diseases with arthroscopy. Chinese Journal of Orthopaedic Surgery, (17):1292-1294.

88. Xu, J., Tian, J., Xu, C. R., Zheng, W. Z., Lyu, S. Q., Tao, S. Q., et al., 2008. Arthroscopic debridement and medullary core decompression combined with autologous peripheral blood stem cell transplantation in the treatment of early avascular necrosis of the femoral head in 38 cases. China Tissue Engineering Research and Clinical Rehabilitation, (03):515-517.
89. Li, Y. P., Zhao, Q. A., Xu, H. P., Shi, F. M., Shi, Z. Y., Liu, Z. X., et al., 2007. Treatment for Osteonecrosis of Femoral Head with Morselized Periosteum and Bone Grafting after Arthroscopic Core Decompression. Chinese Journal of Bone and Joint Injury, (11):902-904.
90. Xiong, H. Z., Deng, Y. H., Jin, Y., Wang, A. H., Hong, S., 2022. An all-arthroscopic light bulb technique to treat osteonecrosis of the femoral head through outside-in fashion without distraction: A case report. Frontiers in surgery, 9, 944480.
91. Palekar, G., Bhalodiya, H. P., Archik, S., Trivedi, K., 2021. Retrospective Study on Implantation of Autologous-Cultured Osteoblasts for the Treatment of Patients with Avascular Necrosis of the Femoral Head. Orthopedic research and reviews, 13, 15-23.
92. Menge, T. J., Briggs, K. K., Rahl, M. D., Philippon, M. J., 2021. Hip Arthroscopy for Femoroacetabular Impingement in Adolescents: 10-Year Patient-Reported Outcomes. The American journal of sports medicine, 49(1), 76-81.
93. Nazal, M. R., Parsa, A., Martin, S. D., 2019. Mid-term outcomes of arthroscopic-assisted Core decompression of Precollapse osteonecrosis of femoral head-minimum of 5 year follow-up. BMC musculoskeletal disorders, 20(1), 448.
94. Dippmann, C., Kraemer, O., Lund, B., Krogsgaard, M., Hölmich, P., Lind, M., et al., 2018. Multicentre study on capsular closure versus non-capsular closure during hip arthroscopy in Danish patients with femoroacetabular impingement (FAI): protocol for a randomised controlled trial. BMJ open, 8(2), e019176.
95. Zhang, H. J., Liu, Y. W., Du, Z. Q., Guo, H., Fan, K. J., Liang, G. H., et al., 2013. Therapeutic effect of minimally invasive decompression combined with impaction bone grafting on osteonecrosis of the femoral head. European journal of orthopaedic surgery & traumatology : orthopedie traumatologie, 23(8), 913-919.
96. Yamamoto, Y., Hamada, Y., Ide, T., Usui, I., 2005. Arthroscopic surgery to treat intra-articular type snapping hip. Arthroscopy : the journal of arthroscopic & related surgery : official publication of the Arthroscopy Association of North America and the International Arthroscopy Association,

21(9), 1120-1125.

97. Hu, C., Wang, T. J., Tang, M. J., Tang, Z. H., 2021. Clinical effect of arthroscopic surgery for hip joint disease. China Disability Medicine,29(6):33-34.

98. Yuan, C. L., Wang, D. M., 2019. Application of Arthroscopic Minimally Invasive Technique in the Treatment of Early Femoral Head Necrosis and its Effect on the Pain Degree of Patients. Heilongjiang Medical Journal, 43(6):587-589.

99. Zhang, Q. X., Zhang, P., Pan, H. L., 2019. A retrospective study of 105 cases of traction-related complications in hip arthroscopy. Chinese Journal of Bone and Joint Surgery,12(2):131-134.

100. Su, L. B., Xiong, K., 2017. Analysis of the efficacy of arthroscopic surgery for the treatment of femoral head necrosis and the key points of surgical treatment. Everybody’s health (middle edition), 11(11):89.

101. Bounds, C. A., Hudson, C. C., 2023. Feasibility of feline coxofemoral arthroscopy using a supratrochanteric lateral portal: A cadaveric study. Veterinary surgery : VS, 52(8), 1202-1208.

102. Espinel Rupérez, J., Serrano Crehuet, T., Hoey, S., Arthurs, G. I., Mullins, R. A., 2023. Arthroscopic-assisted hip toggle stabilization in cats: An ex vivo feasibility study. Veterinary surgery : VS, 52(6), 853-863.

103. Ulfelder, E. H., Hudson, C. C., Beale, B. S., 2019. Correlation of distraction index with arthroscopic findings in juvenile dogs with hip dysplasia. Veterinary surgery : VS, 48(6), 1050-1057.

104. Kim, J., Jeong, J., Lee, H., 2019. Evaluation of a self-retaining distractor for hip joint arthroscopy in toy breed dogs. BMC veterinary research, 15(1), 35.

105. Devesa, V., Rovesti, G. L., Urrutia, P. G., San Roman, F., Rodriguez-Quiros, J., 2014. Evaluation of a joint distractor to facilitate arthroscopy of the hip joint in dogs. The Journal of small animal practice, 55(12), 603-608.

106. Wang, W., Liu, L., Dang, X., Ma, S., Zhang, M., Wang, K., 2012. The effect of core decompression on local expression of BMP-2, PPAR-γ and bone regeneration in the steroid-induced femoral head osteonecrosis. BMC musculoskeletal disorders, 13, 142.

107. Cui, D. P., Zhao, D. W., 2011. Repair of femoral head necrosis by bone marrow mesenchymal stem cells modified with vascular endothelial growth factor/bone morphogenetic protein 2.

Chinese Tissue Engineering Research and Clinical Rehabilitation, 15(01):37-40.

108. Zhang, Y., Tang, J., Yue, Z., Guo, W., Wang, W., 2024. Tension-Free Weight-Bearing Model of Steroid-Induced Osteonecrosis of Femoral Head in Rats. Journal of visualized experiments : JoVE, (211), 10.3791/66883.

109. Zhan, Z. M., Huang, R. T., Chen, Y. F., 2023. Progress in the study of animal models of ischaemic osteonecrosis. International Journal of Orthopaedics, 44(03):146-150.

110. Yang, F., Xia, H. T., Cao, D. G., Yang, F. Y., 2020. Research Progress of Modeling Methods for Avascular Necrosis of Femoral Head. Jiangxi Traditional Chinese Medicine, 51(07):74-77.

111. Ge, Y. J., Cai, M. X., Li, L. P., Chen, H. Y., Pang, Z. H., Fan, Y. G., 2019. Features of animal models of osteonecrosis of the femoral head. Chinese Tissue Engineering Research, 23(35):5690-5696.

112. Wei, Y. J., He, L., Guo, X. R., Cao, L. Z., Zhang, H. L., Zhang, X. G., et al., 2018. Impact of Core Decompression and ShengGu ZaiZao Pills on BMP-2 and VEGF mRNA of Bone Tissue of Rabbit with Steroid-induced Femoral Head Necrosis. Western Traditional Chinese Medicine, 31(09):34-37.

113. Guo, C. L., He, L., Guo, X. R., Cao, L. Z., Yang, X. F., Zhang, H. L., et al., 2018. Effects of Shenggu Zaizao Wan combined with myeloid drilling core decompression in treating steroid induced avascular necrosis of the femoral head on blood lipid level and trabecular architecture in model rabbits. Journal of Gansu University of Traditional Chinese Medicine, 35(01):18-22.

114. Li, M., Li, Z. W., Tang, B. M., Li, Z. Q., Zhang, Y., Ren, R., Hu, Y. B., 2017. Research progress of modeling method for animal model of avascular necrosis of femoral head. Electronic Journal of Integrative Cardiovascular Disease of Chinese and Western Medicine,2017,5(12):4-6.

115. Yu, K. F., Tan, H. B., Xu, Y. Q., 2015. Research progress in the preparation of animal model of ischaemic necrosis of femoral head. Chinese Journal of Repair and Reconstructive Surgery, 29(12):1564-1569.

116. Kelly, B. T., Williams, R. J., 3rd, Philippon, M. J., 2003. Hip arthroscopy: current indications, treatment options, and management issues. The American journal of sports medicine, 31(6), 1020-1037.

117. Yamamoto, Y., Ide, T., Ono, T., Hamada, Y., 2003. Usefulness of arthroscopic surgery in hip trauma cases. Arthroscopy : the journal of arthroscopic & related surgery : official publication of

the Arthroscopy Association of North America and the International Arthroscopy Association, 19(3), 269-273.

118. McCarthy, J., Puri, L., Barsoum, W., Lee, J. A., Laker, M., Cooke, P., 2003. Articular cartilage changes in avascular necrosis: an arthroscopic evaluation. Clinical orthopaedics and related research, (406), 64-70.

119. Yang, X. M., Shi, W., Du, Y. K., Zhang, L., Hu, C. B., Yao, R., et al., 2018. Minimally invasive to treat the early necrosis of femoral head with the application of arthroscopic technique. Biological Orthopaedic Materials and Clinical Research, 15(03):45-49+83.

120. Ji, L. H., Zhao, T. B., Xu, Y., Li, H. H., 2016. The efficacy of arthroscopic surgical treatment for infant with developmental dislocation of the hip. Journal of Clinical Orthopaedics, 19(05):576-577.

121. He, R., Chen, G. X., Yang, L., Guo, L., Duan, X. J., Dai, G., 2013. Short-term clinical results of hip arthroscopy and ambulatory abduction brace for Perthes disease. Chinese Journal of Joint Surgery (Electronic Edition), 7(02):175-180.

122. Luo, G. B., Liao, J., Wu, H., Xiao, Z. M., 2010. Arthroscopic-guided core decompression and bone grafting for early ischaemic necrosis of the femoral head. Journal of Guangxi Medical University, 27(03):431-432.

123. Yang, X. M., Shi, W., Du, Y. K., Zhang, L., 2010. Minimally invasive grafting osteoinductive absorbing material attaching autologous red bone marrow by the decompression of bone marrow core with the assistance of arthroscopy combined with Titanium rod in the treatment of stage II ANFH. Chinese Journal of Clinical Physicians (Electronic Edition), 4(05):622-629.

124. Liu, K., 2009. Analysis of 6 cases of femoral head necrosis treated by hip arthroscopy. Chinese Journal of Misdiagnosis, 9(09):2238-2239.

125. Lyu, Y., Cao, X. Y., 2008. Clinical observation of core decompression combined with hip arthroscopy in the treatment of early ischaemic necrosis of the femoral head. Occupation and Health, (01):87-88.

126. Ji, D. H., Feng, Y., Guo, L., 2008. Surgical coordination of arthroscopic medullary core decompression and autologous stem cell replantation with in vitro culture. Modern Health Care (Medical Innovation Research), 5(9):27-28.

127. Guo, H. S., Ang, L., Liu, X. Z., Liu, F., Liu, G., Liu, H. Z., 2017. Arthroscopic-assisted

marrow core decompression and bone grafting combined with interventional treatment for Ficat stage II femoral head necrosis[J]. Chinese Journal of Bone and Joint Injury, 32(04):390-392.

128. Wang, Z. G., Wang, Y., Liu, Y. J., Li, Z. L., Cai, X., Wei, M., 2007. Clinical study on the treatment of early ischaemic necrosis of the femoral head by small-aperture drilling and decompression and hip arthroscopic cleaning. Chinese Medical Journal, 87(29):2041-2044.

129. Yu, X. Z., Liu, Y. J., Wang, Z. G., Li, Z. L., Zhang, B. X., 2007. Clinical evaluation of early ischaemic necrosis of the femoral head treated with porous core decompression and joint cleaning. Journal of Qingdao University Medical College, (02):97-99.

130. Yu, X. Z., Liu, Y. J., Zhang, B. X., Li, Z. L., Wang, Z. G., Wang, Y., 2007. Clinical evaluation of core decompression of femoral head assisted with arthroscopy debridement for avascular necrosis of femoral head. Journal of the College of Continuing Medical Education, (02):90-91.

131. Zhang, L., Liu, J. S., Sun, J., 2006. Arthroscopic presentation and analysis of femoral head necrosis. Chinese Journal of Minimally Invasive Surgery, (12):912-913+923.

132. Zhang, X. S., Chen, X. M., Li, Z. H., Zhang, Q., Chen, Y., Peng, D., et al., 2006. Arthroscopy for the diagnosis and treatment of the hip joint disease in children. Journal of Clinical Orthopaedics, (01):22-24.

133. Wang, K. L., Zhuang, Y., Dai, G. F., Liu, Y. Z., 2005. Arthroscopic treatment for developmental dislocation of the hip in children. Chinese Journal of Paediatric Surgery, (11):565-566.

134. Liu, Y. J., Wang, Y., Li, Z. L., Cai, X., Wang, Z. G., 2005. The value of hip arthroscopic clearance of small-diameter multiorifice multidirectional intramedullary decompression in the treatment of femoral head necrosis. Chinese Journal of Orthopaedic Surgery, (15):1141-1143.

135. Zhang, J., Liu, Y., 2005. Nursing care of arthroscopic medullary decompression access BMP/bovine cancellous bone complex implantation for the treatment of ischaemic necrosis of the femoral head. Chinese Journal of Misdiagnosis, (08):1546-1547.

136. Zhao, D. W., Wang, B. J., 2004. Arthroscopic application of bone flap transfer with vascular tibia for the treatment of ischaemic necrosis of femoral head. Chinese Journal of Microsurgery, (04):17-18.

137. Xu, J. Z., Fang, D. W., Zhang, W. X., 2004. Comprehensive treatment of early ischaemic necrosis of femoral head by hip arthroscopy. Chinese orthopedics, (07):23-24.

138. Sun, K., Liang, B., Wei, M. K., 2004. Report of a case of hip arthroscopy for the treatment of hip joint lesion. Guangxi medicine, (12):1855-1856.
139. Chen, W. H., Zhang, L., Liu, D. B., Zhang, Q., Zhang, H. M., Zhao, T. J., et al., 2004. Clinical study of combined minimally invasive surgery for femoral head necrosis. Chinese Journal of Orthopaedic Surgery, (17):6-8.
140. Li, Z. L., Wang, Y., Liu, Y. J., Wang, Z. G., Cai, X., 2003. A preliminary report on the treatment of early aseptic necrosis of the femoral head by drilling and decompression under hip arthroscopy. Chinese Journal of Orthopaedic Surgery, (24):34-36.
141. Zhao, D. W., Wang, W. M., Cui, X., 2001. Treatment of ischaemic necrosis of the femoral head by arthroscopic transfer of a large rotor flap with vascular tibia. Chinese Journal of Microsurgery, (04):4-6.
142. Liu, Y. J., Li, Z. L., Wang, Z. G., Yuan, X. C., Wang, Y., 2002. Application of arthroscopy in the diagnosis and treatment of hip joint disorders. Chinese Journal of Surgery, (12):35-38.
143. Yang, J., Sun, P., Liu, Z., Li, Y., Zhang, J., Liu, Y., et al., 2024. Mid-term Clinical Outcomes of "Light Bulb" Core Decompression with Arthroscopic Assistance in Peri-collapse Osteonecrosis of the Femoral Head: A Retrospective Comparative Study. Orthopaedic surgery, 16(6), 1399-1406. https://doi.org/10.1111/os.14058
144. Zhao, G., Liu, Y., Zheng, Y., Wang, M., Li, Z., Li, C., 2024. Hip Arthroscopy Debridement Combined with Multiple Small-Diameter Fan-Shaped Low-Speed Drilling Decompression in the Treatment of Early and Middle Stage Osteonecrosis of the Femoral Head: 14 Years Follow-Up. Orthopaedic surgery, 16(3), 604-612.
145. Zhao, Y., Zhang, G., Song, Q., Fan, L., Shi, Z., 2023. Intramedullary core decompression combined with endoscopic intracapsular decompression and debridement for pre-collapse non-traumatic osteonecrosis of the femoral head. J Orthop Surg Res, 18(1), 6.
146. Lian, K. Q., 2021. Effect of arthroscopic compression graft fibular support on hip function after peri-collapse non-traumatic femoral head necrosis. Journal of Shanxi Health and Health Professions College, 31(02):26-28.
147. Dou, T., Suo, N. A. X., Guo, Q., Wang, J., 2020. Arthroscopic debridement combined with multiple decompressions for avascular necrosis of femoral head. Orthopedic Journal of China, 28(17):1558-1562. doi: 10.3977/j.issn.1005-8478.2020.17.05.

148. Zhang, Y., Fang, H. L., 2020. Arthroscopically monitored compression grafting fibular support for non-traumatic femoral head necrosis in the peri-collapse phase. Henan Medical Research, 29(36):6769-6771. doi:10.3969/ j.issn.1004- 437X.2020.36.016.

149. Li, J., Li, Z. L., Zhang, H., Su, X. Z., Wang, K. T., Yang, Y. M., 2017. Long-term Outcome of Multiple Small-diameter Drilling Decompression Combined with Hip Arthroscopy versus Drilling Alone for Early Avascular Necrosis of the Femoral Head. Chin Med J, 130(12), 1435-1440.

150. Li, J., Li, Z. L., Su, X. Z., Liu, C. H., Zhang, H., Wang, K. T., 2017. Effectiveness of multiple small-diameter drilling decompression combined with hip arthroscopy for early osteonecrosis of the femoral head. Chinese Journal of Reparative and Reconstructive Surgery, 31(09):1025-1030.

151. Zhuang, Z. K., Wu, Z. K., Xie, Q. H., Liu, X. H., Zhang, H. T., Sun, R. B., et al., 2017. Arthroscopic treatment of peri-collapsed femoral head necrosis with compression grafting and fibular support. Shandong Medicine, 57(05):59-61. doi: 10.3969 / j.issn.1002-266X.2017.05.019.

152. Liu, G., 2015. linical Efficacy of Core Decompression Combined with Implantation of Artificial BAM Bone by Using Arhtroscopy in the Treatment of Ischemic Necrosis of Femoral Head. Journal of Guiyang Medical College, 40(11):1256-1258+1261.

153. Wu, G., Zhang, Y. F., 2015. Arthroscopic core decompression with autologous bone marrow induction material combined with titanium rod in the repair of stage II femoral head necrosis. Chinese Journal of Tissue Engineering Research, 19(28):4460-4464.

154. Liu, B. Y., Zhao, D. W., Guo, L., Yang, L., Wang, B. J., Fu, W. M., 2013. Outcome of the core decompression with arthroscopy in treating early - stage osteonecrosis of femeral head. Journal of Hebel Medical University, 34(06):646-648.

155. Zhuo, N. Q., Wan, Y. X., Lu, X. B., Zhang, Z. J., Tian, M. Y., Chen, G., 2012. Comprehensive management of early stage avascular necrosis of femoral head by arthroscopic minimally invasive surgery. Chinese Journal of Reparative and Reconstructive Surgery, 26(09):1041-1044.

156. Han, Z., Tao, M., Zhang, Z., Zheng, J. H., 2008. Surgical treatment of necrosis of the femoral head in early stage with core decompression and arthroscopy debridement. Shandong Medicine, 48(40):37-39.
